# Chemotaxonomic Monitoring of Genetically Authenticated Amomi Fructus Using High-Performance Liquid Chromatography–Diode Array Detector with Chemometric Analysis

**DOI:** 10.3390/molecules25194581

**Published:** 2020-10-07

**Authors:** Eui-Jeong Doh, Guemsan Lee, Hyun-Jong Jung, Kang-Beom Kwon, Jung-Hoon Kim

**Affiliations:** 1 Research Center of Traditional Korean Medicine, Wonkwang University, Iksan 54538, Korea; bluemoonlion@wku.ac.kr; 2 Department of Herbology, College of Korean Medicine, Wonkwang University, Iksan 54538, Korea; rasfin@wku.ac.kr; 3 Department of Diagnostics, College of Korean Medicine, Wonkwang University, Iksan 54538, Korea; kendu@wku.ac.kr; 4 Department of Korean Medicinal Physiology, College of Korean Medicine, Wonkwang University, Iksan 54538, Korea; desson@wku.ac.kr; 5 Division of Pharmacology, School of Korean Medicine, Pusan National University, Yangsan 50612, Korea

**Keywords:** Amomi Fructus, phylogenetic identification, HPLC chromatographic profiling, chemometric analysis, chemotaxonomic monitoring

## Abstract

Amomi Fructus is widely used to treat digestive disorders, and *Amomum villosum*, *A. villosum* var. *xanthioides*, and *A. longiligulare* are permitted medicinally in national pharmacopeias. However, there are a variety of adulterants present in herbal markets owing to their morphological similarities to the genuine *Amomum* species. Forty-two Amomi Fructus samples from various origins were identified using internal transcribed spacer and chloroplast barcoding analyses, and then their chromatographic profiles were compared using chemometric analysis for chemotaxonomic monitoring. Among the Amomi Fructus samples, *A. villosum*, *A. longiligulare*, *A. ghaticum*, and *A. microcarpum* were confirmed as single *Amomum* species, whereas a mixture of either these *Amomum* species or with another *Amomum* species was observed in 15 samples. Chemotaxonomic monitoring results demonstrated that two medicinal *Amomum* samples, *A. villosum* and *A. longiligulare*, were not clearly distinguished from each other, but were apparently separated from other non-medicinal *Amomum* adulterants. *A. ghaticum* and *A. microcarpum* samples were also chemically different from other samples and formed their own species groups. *Amomum* species mixtures showed diverse variations of chemical correlations according to constituent *Amomum* species. Genetic authentication-based chemotaxonomic monitoring methods are helpful in classifying Amomi Fructus samples by their original species and to distinguish genuine *Amomum* species from the adulterants.

## 1. Introduction

Amomi Fructus (Sa-In) has been used to treat digestive disorders associated with excessive dampness and originates from the ripe fruits or seed clusters of *Amomum villosum* Lour. and *A. villosum* var. *xanthioides* (Wall. ex Baker) T.L. Wu and S.J. Chen in the Korean pharmacopeia [1], whereas the ripe fruit of *A. longiligulare* T.L. Wu including those of the two species mentioned is additionally registered in the Chinese pharmacopeia [2]. However, the commercial status of Amomi Fructus in herbal markets does not correspond to official documents; that is, unofficial *Amomum* species or even *Alpinia* species are often sold and used as counterfeits or adulterants of Amomi Fructus [3,4].

This is because the fruits of *A. villosum* are recognized as being of high commercial grade among official *Amomum* species and consequently, are sold at a high price in herbal markets. Particularly, the indistinguishable organoleptic properties among official *Amomum* species and the adulterants exacerbate the misuse of Amomi Fructus. Owing to their morphological similarity, Amomi Fructus from different *Amomum* species cannot be clearly discriminated through macroscopic observation, which creates confusion in their medicinal application. Therefore, more accurate and precise techniques using genetic information have been developed to identify Amomi Fructus from other counterfeits. Internal transcribed spacer (ITS) and *matK* DNA sequencing [5], nuclear ribosomal DNA (nrDNA) ITS1 sequencing [6], genomic DNA barcoding using ITS [3] or single nucleotide polymorphism (SNP) genotypes [7], and chloroplast genome sequencing [8,9,10] have been used to identify various *Amomum* species at the genetic level.

Chemotaxonomic monitoring of herbal medicines using the chromatographic profiling method is another approach to identify and distinguish Amomi Fructus from various *Amomum* species. As Amomi Fructus is chemically characterized by abundant volatile oil, gas chromatography–mass spectrometry (GC–MS) has mainly been used to determine chemical constituents in the volatile oil from *Amomum* species [11,12,13,14]. Furthermore, Amomi Fructus samples originating from *A. villosum* and *A. villosum* var. *xanthioides* were chemically distinguished by the composition of their volatile oils using GC–MS combined with chemometric techniques [4]. However, the chemotaxonomic approach based on volatile constituents is insufficient to guarantee the chemical features of Amomi Fructus as these oils are easily vaporized when extracted at high temperature. Non-volatile constituents in Amomi Fructus remain after hot extraction and hence, these can possibly exert therapeutic activity when administered to patients. Previous studies reported the isolations of non-volatile constituents: flavonoids (quercetin, quercitrin, and isoquercitrin); phenolic acids (vanillic acid and 3,4-dihydroxy-benzoic acid), coumarins (flavanocoumarin and isoflavanocoumarin), and steroids (daucosterol, stigmasterol, and ergosterol) [15,16]. Consequently, chemical characterization using non-volatile constituents that are determined using high-performance liquid chromatography (HPLC) is also crucial to evaluate the chemotaxonomic discrimination of *Amomum* species. Several studies have reported the chemical discrimination of *A. villosum* and its adulterants using chromatographic fingerprinting with HPLC-based statistical analysis [17,18]. Although there is a limitation that Amomi Fructus samples used in those studies were not clearly authenticated before the HPLC experiment, chemical differences were observed.

To overcome the inaccuracy of herbal samples in chemotaxonomic analysis, a process of ‘genetic authentication coupled chromatographic profiling’ is required to support chromatography-based chemotaxonomic results. Currently, chemical analysis combined with DNA barcoding techniques is recognized as a powerful tool for identification and differentiation in the chemotaxonomy of herbal medicines [19,20]. Recent studies reported that the combination of DNA barcoding and HPLC fingerprinting was applied to identify *Salvia* species [21], *Phellodendron* species [22], and *Daphne* species [23]. Our research group also established a ‘genetic authentication-coupled chromatographic profiling’ process to chemically distinguish *Atractylodes* species using ITS sequencing, HPLC analysis, and chemometric tools [24,25]. Therefore, we collected 42 Amomi Fructus samples and genetically authenticated these using ITS sequences from nrDNA and chloroplast genome-based DNA barcoding analysis. A HPLC–diode array detector (HPLC–DAD) was used to construct the chromatographic profiles for chemical monitoring of Amomi Fructus samples.

## 2. Results

### 2.1. ITS Regions of the Nuclear Ribosomal Cistron

To identify the species of the 42 Amomi Fructus samples, the nucleotide sequences of the ITS region were analyzed. Approximately 645–665 bases of amplified product sequence were identified based on the samples listed in Table 1 and Table 2. As shown in our previous study [3], several adulterants were also observed in this study. Especially this time Amomi Fructus samples were collected from diverse countries where they were used for not only medical purposes but also as food products. Therefore, various adulterants of *Amomum* species were identified and some of these were mixed with each other, and especially, the mixture of species was mostly shown in the samples from Southeast Asia (Table 2). Most of the samples from China were identified as *A. villosum* or *A. longiligulare*, except for two samples, AGS01 (*A. ghaticum*) and AMS04 (*A. microcarpum*). In contrast, NCBI BLAST analysis confirmed *A. ghaticum*, *A. microcarpum*, *A. uliginosum,* and *A. longiligulare* in the samples from Myanmar. In addition, *A. microcarpum*, *A. longiligulare*, *A. villosum*, *A. tomrey* var. *tomrey*, *A. koenigii*, *A. chinense,* and *A. echinocarpum* were also observed. The sequence identity matrix ranged from 0.989 to 0.883 in adulterants of *Amomum*, whereas it ranged from 0.885 to 0.841 in adulterants of *Alpinia* (Table S1, Figure S1).

### 2.2. Chloroplast Genome-Based DNA Barcode Sequence Analysis

According to the previous study, three chloroplast genome areas (*rbcL*, *matK*, and *trnL-F* intergenic spacer) were used as support for the identification result of the ITS region [3]. The *psbA-trnH* region was additionally analyzed to identify adulterant *Amoum* species in the work. Sequence characteristics of the four plastid regions and one nuclear region are presented in Table 3. The *rbcL* and *trnL-F* intergenic spacer regions showed higher variable sites among the four plastid regions (except the ITS region). The sequences of *matK* and *psbA-trnH* were the most conserved among the five regions analyzed when the aligned length and number of conserved sites were considered. Even though the variable sites of the four plastid loci were much lower than those of the ITS region and highly conserved, they could separate the Amomi Fructus species from those of the adulterants in *Amomum* and *Alpinia* and the results were consistent with the analysis of the ITS region.

For more details regarding the four plastid loci, partial nucleotide sequences of the 933 bases in *matK* were determined using the 390F/1326R primer set. Furthermore, the sequence identity matrix between Amomi Fructus and the adulterants was considerably closer than the results with ITS; i.e., the minimum identity matrix result was 0.979 between Amomi Fructus and the adulterants including *Alpinia* (Table S1). Although the *matK* could not distinguish between *A. villosum* and *A. uliginosum*, it could classify the rest of species in this work.

For the *rbcL* gene sequence, we determined partial nucleotide sequences of 743 bases in all the samples listed in Table 1 and Table 2 using the rbcL a-f/724R primer set. The number of variable sites in the nucleotide sequence was lower than that in the ITS results and some of the adulterants in *Amomum* shown identical results; however, the three genuine species of Amomi Fructus could still be distinguished as shown in the previous study [3]. The minimum sequence identity matrix of *rbcL* among samples in Table 1 and Table 2 was nearly 0.900 (except for the Amomi Tsao-ko Fructus samples whose minimum sequence identity matrix was 0.897).

For the *psbA-trnH* intergenic spacer, partial nucleotide sequences of 800–836 bases were determined using the trnH2/psbAF primer set. Although the *psbA-trnH* intergenic spacer showed the most conserved sites with the *matK* area, the sequence length was different depending on the species. Thus, the sequence identity matrix range was 0.997–0.914 and well classified the species of Amomi Fructus from several adulterants as was observed with the ITS region.

In the case of the *trnL-F* intergenic spacer, amplified products with 395–415 bases were determined from the samples listed in Table 1 and Table 2. The amplified product size was different depending on the species and the sequence identity matrix between Amomi Fructus and the adulterants ranged from 0.990 to 0.88.

### 2.3. Phylogenetic Analysis

**Figure 1:**
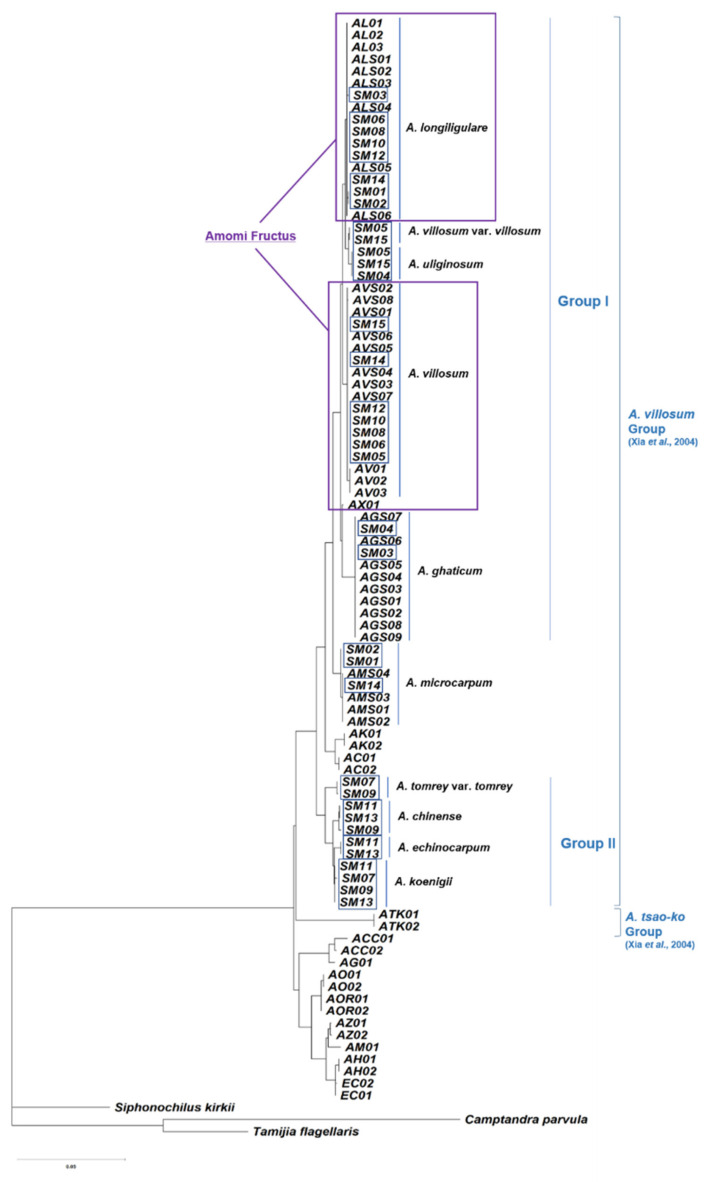
Maximum likelihood-based inference of phylogenetic tree with smart model selection constructed based on concatenated nucleotide sequences of an ITS (internal transcribed spacer) and four chloroplast genome-based DNA barcode regions. AX01: *Amomum villosum* var. *xanthioides* nucleotide sequence of KJ151892 and MH161417. The box indicates the species-mixed samples.

The PhyML+SMS tree was constructed based on the concatenated nucleotide sequences of the ITS and the four chloroplast genome-based DNA barcode regions (Figure 1). The species in the phylogenetic tree were well separated by their original species, which supported the accuracy of the identification result based on the ITS region. The adulterants derived from *Alpinia* were divided into different clades distant from the plants in the *Amomum* group. Among the species in *Amomum*, three original species of Amomi Fructus, *A. longiligulare*, *A. villosum,* and *A. villosum* var. *xanthioides* were in group I and *A. ghaticum*, *A. villosum* var. *villosum,* and *A. uliginosum* were grouped very close together. *A. microcarpum* was also located close to group I. In addition, *A. tomrey* var. *tomrey*, *A. chinense*, *A. echinocarpum,* and *A. koenigii* were assembled close together in group II.

### 2.4. Optimization of Sample Extraction and Analytical Conditions

The extraction of the representative Amomi Fructus sample (ALS01 in Table 1) was performed using the rapid, efficient, and simple ultrasonic extraction method. Extraction times (10, 30, and 50 min) and solvent ratios (methanol:water = 1:0, 0:1, and 1:1) were compared and the extraction for 30 min with solvent mixture (methanol:water = 1:1) was selected to obtain a higher absolute area of the selected peaks in the chromatogram of ALS01.

HPLC analytical conditions were optimized in terms of the mobile phase modifier, mobile phase composition, and UV detection wavelength. Water with 0.1% formic acid (*v*/*v*), water with 0.1% TFA (*v*/*v*), and water without a modifier were compared with various mobile phase ratios of acetonitrile and water. Water containing 0.1% TFA as the aqueous mobile phase produced better interpeak separations and peak shapes with higher peak areas. Six detection wavelengths were selected in the DAD according to the optimal UV absorbance of each peak: 11 peaks at UV 225 nm, 7 peaks at UV 265 nm, 16 peaks at UV 280 nm, 9 peaks at UV 290 nm, 18 peaks at UV 310 nm, and 16 peaks at UV 320 nm (Table S2). Peak No. 18 and peak No. 35 were identified as vanillic acid and quercitrin, respectively, by comparison of their retention times and UV detection wavelengths with those of purchased standard compounds.

Intraday precisions of ALS01 were <0.2% and <8.5%, and interday precisions were <2.0% for retention time and <9.0% for retention time and peak area, respectively (Table S3).

### 2.5. Chromatographic Profiling of Amomi Fructus Samples

**Figure 2:**
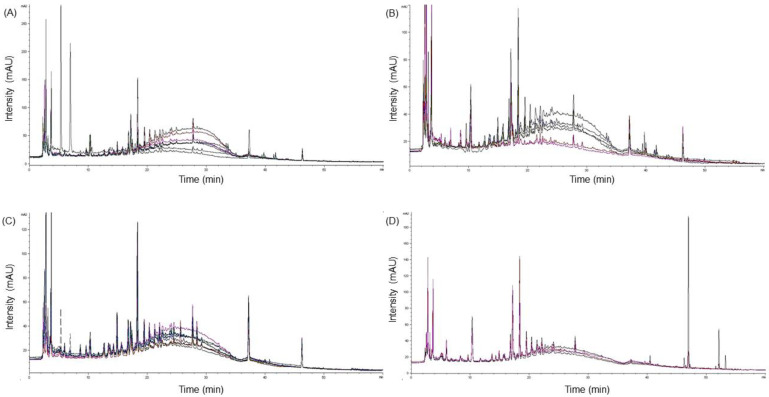
Overlapped chromatograms of the single species of Amomi Fructus samples at 280 nm of detection wavelength (diode array detector (DAD)). (**A**), *Amomum villosum* samples (AVS01–08); (**B**), *A. longiligulare* samples (ALS01–06); (**C**), *A. ghaticum* samples (AGS01–09); and (**D**), *A. microcarpum* samples (AMS01–04).

Overlapped chromatograms of Amomi Fructus samples were mostly similar within interspecies and between interspecies of *Amomum*, with the exception of AVS02 within *A. villosum* samples, and *A. microcarpum* samples among other *Amomum* species, particularly after 40 min of retention time (Figure 2). Chromatograms of Amomi Fructus samples mixed with more than two *Amomum* species showed similar chromatographic patterns according to those of their original species; however, the species-mixed samples whose original species was not obtained in this study (SM07, -09, -11, and -13) showed distinguishable chromatographic patterns from those of the *Amomum* samples listed above (Figures S2 and S3).

There were a number of peaks that showed significant differences in average peak areas between two Amomum species: 6 peaks between *A. villosum* and *A. longiligulare* samples (peak 27, 40, 47, 54, 74, and 77), 15 peaks between *A. villosum* and *A. ghaticum* samples (peak 3, 4, 8, 13, 18, 21, 27, 30, 31, 40, 42, 43, 59, 64, and 77), 14 peaks between *A. villosum* and *A. microcarpum* samples (peak 2, 3, 12, 18, 21, 25, 27, 30, 32, 35, 40, 43, 75, and 77), 9 peaks between *A. longiligulare* and *A. ghaticum* samples (peak 4, 8, 13, 14, 18, 21, 31, 40, and 64), 11 peaks between *A. longiligulare* and *A. microcarpum* samples (peak 2, 3, 12, 18, 25, 27, 36, 47, 59, 75, and 77), and 14 peaks between *A. ghaticum* and *A. microcarpum* samples (peak 2, 4, 8, 11, 13, 14, 18, 19, 27, 31, 43, 59, 75, and 77; Figure S4).

### 2.6. Clustering Analysis of Amomi Fructus Samples Using Chemometric Statistical Methods

Chemotaxonomic classification of Amomi Fructus samples was estimated using chemometric clustering tools with principle component analysis, hierarchical clustering analysis (HCA), and heatmap analysis, and Pearson’s correlation coefficients were calculated to investigate the chemical relationship between the Amomi Fructus samples.

**Figure 3:**
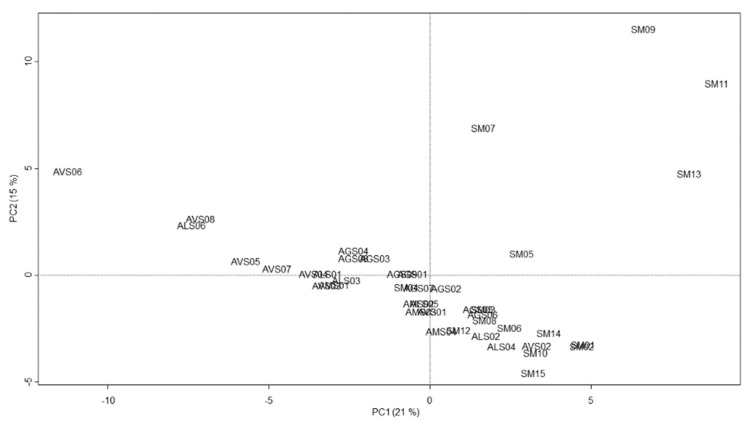
Score plot of principal components (PC1 vs. PC2) on the variables (absolute area of reference peaks) with Amomi Fructus samples. PC1 and PC2 represent 21% and 15% of the total variance, respectively. PC, principal component.

In the principal component (PC) plot, the samples of single *Amomum* species did not form distinct clusters separately by their original species; i.e., those samples were not clearly grouped, as single species samples and species-mixed samples were colocated within the middle range of the PC1 and PC2 scores. However, one *A. villosum* sample (AVS06) and four species-mixed samples (SM07, -09, -11, and -13) were exceptionally apart from the samples distributed together by their wide ranges in PC1 and PC2 scores (Figure 3).

**Figure 4:**
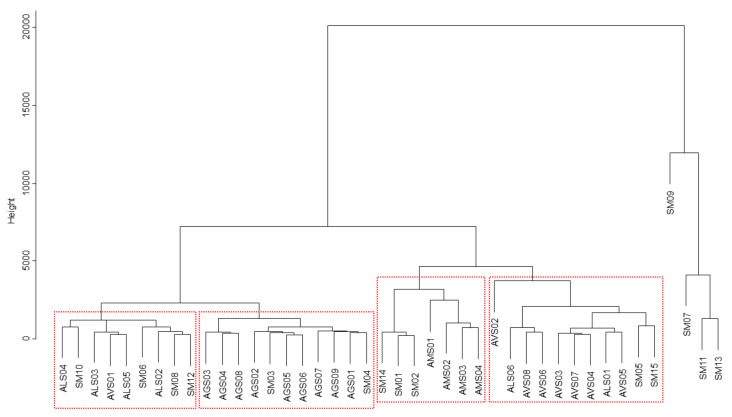
Dendrogram of Amomi Fructus samples from the hierarchical clustering analysis.

The dendrogram from the HCA showed distinct clusters of Amomi Fructus samples according to their original *Amomum* species. One *A. villosum* sample (AVS01) and two *A. longiligulare* samples co-existed exclusively in the *A. longiligulare* group and *A. villosum* group, respectively. In contrast, *A. ghaticum* and *A. microcarpum* samples formed their original cluster groups without the coexistence of other species. Like in the PC plot, four species-mixed samples (SM07, -09, -11, and -13) were unambiguously clustered in a separate group (Figure 4).

**Figure 5:**
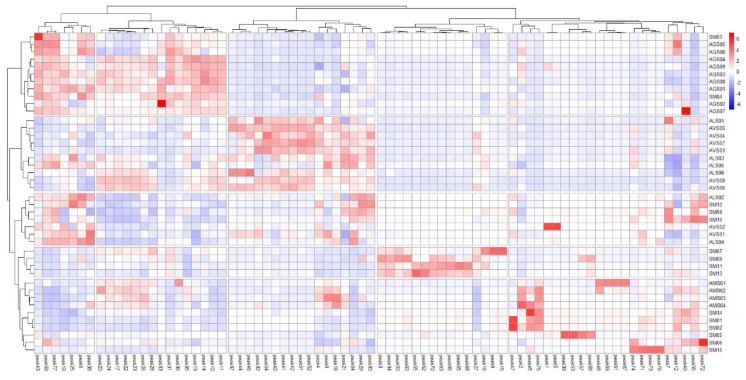
Clustered heatmap of Amomi Fructus samples and profiling peaks.

In the cluster-based heatmap analysis, apparent clusters of Amomi Fructus samples were also observed by their species and the classification of single *Amomum* species samples were analogous to those in the dendrogram of HCA: two *A. villosum* + *A. longiligulare* groups, an *A. ghaticum* group, and an *A. microcarpum* group. Exceptionally, some samples of *A. villosum* and *A. longiligulare* were differently clustered in between the *A. villosum* + *A. longiligulare* groups, compared with the HCA dendrogram (AVS02 moved in, and ALS03 and -05 moved out). Such a shift was also observed in the species-mixed samples (SM05, -06, and -15). Consistent with the principle component analysis and HCA results, SM07, -09, -11, and -13 samples were also obviously distinguished (Figure 5).

### 2.7. Similarity Evaluation of Amomi Fructus Samples Using the Pearson’s Correlation Coefficient

**Figure 6:**
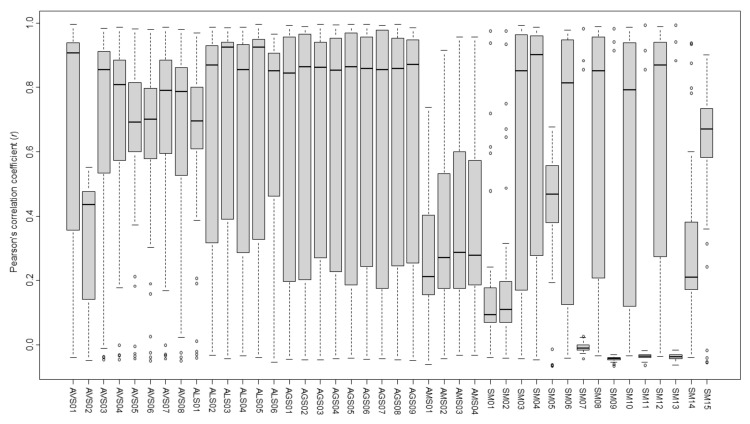
Average coefficients of the Pearson’s correlation coefficient of Amomi Fructus samples.

Correlation between individual Amomi Fructus samples was measured using the Pearson’s correlation coefficient (*r*; Table S4). The ranges of the mean and median values of the coefficients between each Amomi Fructus sample and the remaining samples (rest) were as follows: *A. villosum* samples (AVS) to the rest, 0.336–0.696 mean, and 0.436–0.907 median; *A. longiligulare* samples (ALS) to the rest, 0.629–0.698 mean, and 0.695–0.924 median; *A. ghaticum* samples (AGS) to the rest, 0.657–0.674 mean, and 0.844–0.871 median; *A. microcarpum* samples (AMS) to the rest, 0.271–0.368 mean, and 0.212–0.278 median; and species-mixed samples (SM) to the rest, 0.030–0.686 mean, and -0.042–0.902 median (Figure 6).

Moreover, the results of mean and median *r* values showed that the intraspecies relation was AGS–AGS > ALS–ALS > AVS–AVS ≒ AMS–AMS, whereas interspecies relation was ALS–AGS > AVS–ALS > AVS–AGS > AVS–AMS > ALS–AMS > AGS–AMS. AVS02 showed an exceptionally low correlation to other samples. The correlations of SM to single *Amomum* species were weaker than those of other intra- and interspecies samples owing to lower mean and median *r* values (< 0.5), particularly with wide variations (Table 4, Figures S5–S9).

## 3. Discussion

Amomum is currently recognized as the second largest genus in Zingiberaceae with approximately 150–180 species [5,26,27]. The distribution area of *Amomum* is from Sri Lanka to the Himalayas, China, Southeast Asia, Malaysia, and Northern Australia. Especially, the forests of Southeast Asia are treated as the center of endemism [28]. It suggests there is a possibility that another species of adulterant may be found, such as *A. ghaticum*, a newly found species within Amomi Fructus in our previous study [3].

In the 42 Amomi Fructus samples collected in this study, we also identified several adulterant species in *Amomum* and some of these were not previously mentioned as an adulterant of Amomi Fructus in the literature. The typical examples were *A. uliginosum* (SM04, SM05, and SM15), *A. ghaticum* (AGS01–AGS09, SM03, and SM04), *A. tomrey* var. *tomrey* (SM07 and SM09), and *A. echinocarpum* (SM11 and SM13). Additionally, seven *A. microcarpum* (AMS01–04, SM01, SM02, and SM14), four *A. koenigii* (SM07, SM09, SM11, and SM13), and three *A. chinense* (SM09, SM11, and SM13) were found in this study and they are recoded as ‘Se-Sa-In (xi sha ren)’, ‘Ya-Cho-Gwa (ye cao guo)’, and ‘Hae-Nam-Ga-Sa-In (hai nan jia sha ren)’ in the Flora of China. SM05 and SM15, identified as *A. villosum* var. *villosum* (KJ151892 and MH161417) through the NCBI BLAST analysis, are recorded as autonyms of *A. villosum* in the Flora of China. *A. uliginosum* is a widespread species in Laos, Cambodia, Vietnam, Thailand, Peninsular Malaysia, and the Sumatra area, and is very similar to *A. villosum* (continental Southeast Asia) in the shape of the flowering head and the small, dark-red, prickly fructus [28]. They showed similarities not only in the morphological feature, but also in the close sequence identity matrix in all five DNA barcode regions. The results of the phylogenetic analysis inferred through ITS nucleotide sequences showed that samples of genuine *Amomum* species formed a closer relationship and their groups were apparently distinguished from the other *Amomum* and *Alpinia* species, which were recognized as adulterants. Our results also support the clades of taxa in the Alpinioideae that include species of *Amomum* as proposed by the previous study [5].

Chemotaxonomic relevance among *Amomum* species was presented using chromatographic profiling combined with chemometric analysis and chemical characteristics of samples were obviously differentiated by their own species. The concordance rate of clustering single *Amomum* species samples into each independent species group in HCA was 87.5%, 66.7%, and 100% for samples of *A. villosum*, *A. longiligulare*, and both *A. ghaticum* and *A. microcarpum*, respectively. Particularly, *A. villosum* and *A. longiligulare* samples medicinally permitted in the Korean and Chinese pharmacopeias were chromatographically analogous to each other and they were distinctly separated from the other non-medicinal *Amomum* species in the cluster analysis. Although most *A. villosum* samples were distinguished from *A. longiligulare* samples, a few *A. villosum* or *A. longiligulare* samples interrupted the apparent distinction between the two species; i.e., AVS01, –02 and ALS01, –06 showed higher correlations with the opposite species. *A. villosum* and *A. longiligulare* samples are genetically divided into their own *Amomum* species in this study as well as in a previous study [8]; however, the chemical similarities between the samples of these two species makes for an ambiguous classification using chemometric cluster analyses, as reported previously [18].

*A. ghaticum*, a species mainly occurring in the Western Ghats of India, mostly originated from Myanmar and was confirmed as a major adulterant species among genuine Amomi Fructus in local herbal markets in this and a previous study [3]. The samples of *A. ghaticum* obviously formed their own species group in the cluster analysis, indicating that this species represented distinguishable chromatographic profiles from other *Amomum* species, notwithstanding their macroscopic similarities. However, the correlation analysis produced contradictory results that *A. ghaticum* samples showed comparatively high correlation coefficients (*r*) with *A. villosum*, and especially, *A. longiligulare* samples, meaning strong linear relationships between the chromatographic profiles of *A. ghaticum* and those of the above two species [29]. In contrast, *A. microcarpum* samples were genetically and chemically distant from the above three *Amomum* species, as shown from chromatographic patterns, cluster analysis, and correlation coefficients.

Interestingly, species-mixed samples, when sharing at least one of the original species in tested samples, were correspondently included into a single *Amomum* species group; for example, SM08, –10, and –12 were grouped in AVS–ALS clusters. In contrast, those samples without any sharing among the four *Amomum* species exclusively separated from the other samples, having extremely low correlations; for example, SM07, –09, –11, and –13, which included *A. tomrey* var. *tomrey*, *A. koenigii*, *A. chinense*, and *A. echinocarpum*, were grouped in their own cluster and their correlation coefficients in comparison to those of other samples were near ‘0′ or even negative. These results demonstrated that these species-mixed samples are phylogenetically distant from the medicinal *Amomun* species and are also exclusively distinguishable by chemical analysis.

Taken together, we found that there were large quantities of Amomi Fructus mixed with non-medicinal adulterants of *Amomum* in herbal markets. Higher chemical correlations and morphological resemblance might cause serious confusion in the Amomi Fructus trade, and consequently, can result in unintended and undesirable effects on patients and health systems. Chemotaxonomic monitoring based on phylogenetic authentication can exclude medicinally non-available adulterants from the genuine Amomi Fructus. Moreover, it is effective in corroborating morphology-based inspection and is essentially required to improve the quality of medicinally available *Amomum* species.

Despite this distinct classification of Amomi Fructus, we had a few limitations in this study: (1) insufficient numbers of Amomi Fructus samples per single *Amomum* species owing to difficulties in collecting genuine *Amomum* species and a mixture of diverse adulterants; (2) the absence of another medicinally available Amomi Fructus, *A. villosum* var. *xanthioides*, owing to its scarcity in local markets or natural habitats; and (3) a lack of chemical research on *Amomum* species other than *A. villosum*.

## 4. Materials and Methods

### 4.1. Plant Materials and Reagents

Methanol, water, and acetonitrile (HPLC grade) were purchased from J.T. Baker (Phillipsburg, NJ, USA). Trifluoroacetic acid (TFA) and formic acid were purchased from Sigma-Aldrich (St Louis, MO, USA). Vanillic acid (peak No. 18) and quercitrin (peak No. 35) were purchased from Fluka (AG, Buch/SG, Switzerland) and ChemFace (Wuhan, Hubei, China), respectively.

Forty-two samples of Amomi Fructus were collected from their natural habitats, agricultural fields, and local markets in Korea, China, Myanmar, and Vietnam, and were also provided by the Korea Institute of Oriental Medicine (Table 1). For species identification of Amomum Fructus, the vouchers of Amomi Fructus and adulterants identified previously [3] were used for comparison with the newly collected samples in this study (Table 2). All the samples and isolated genomic DNA have been deposited at the herbarium of the college of Korean Medicine in Wonkwang University.

### 4.2. Preparation of Genomic DNA

The genomic DNA was extracted from Amomi Fructus samples according to the NucleoSpin^®^ Plant II kit manual (Macherey-Nagel, Düren, Germany) with PL1 lysis buffer. For some samples, 10% cetyltrimethyl ammonium bromide and 0.7 M NaCl were used to remove the phenolic compounds and polysaccharides.

### 4.3. Polymerase Chain Reaction (PCR) Amplification for DNA Barcode Analysis

For ITS amplification, PCR was performed using a T-personal cycler (Biometra, Jenam Germany). In brief, 600 nM of the primer set of ITS1 (5′′-TCCGTAGGTGAACCTGCGG-3′) and ITS4 (5′-TCCTCCGCTTATTGATATGC-3′) [30], 1X AccuPower^®^ GoldHotStart Taq PCR PreMix (Bioneer, Daejeon, Korea), and 30 ng of genomic DNA were used for PCR amplification. PCR cycling followed a predenaturation process (95 °C, 5 min) and the conditions were as follows: denaturation process (95 °C, 30 s), annealing process (52 °C, 30 s), extension process (72 °C, 40 s) × 36 cycles, and final extension process (72 °C, 5 min). For chloroplast DNA barcoding regions, rbcL a-f (5′-ATGTCACCACAAACAG AGACTAAAGC-3′)/724R (5′-TCGCATGTACCTGCAGTAGC-3′) and 390F (5′-CGATCTATTCATTCAATATTT C-3′)/1326R (5′-TCTAGCACACGAAAAGTCGAAGT-3′) primer sets were used for amplification of the rbcL and matK regions [31,32,33]. trnL-e (5′-GGTTCAAGTCCCTCTTATCCC-3′)/trnL-f (5′-ATTTGAACTGGTGACACGAG-3′) and trnH2 (5′-CGCGCATGGTGGATTCACAATC C-3′)/psbAF (5′-GTTATGCATGAACGTAATGCTC-3′) primer sets were used for trnL-F intergenic spacer and psbA-trnH intergenic spacer regions [34,35,36]. The amplified PCR products were separated using 1.5% agarose gel electrophoresis after staining with Safe-White^TM^ (ABM Inc., Richmond, BC, Canada).

### 4.4. Determination of DNA Sequences of PCR Product

PCR products separated from agarose gel were cloned using the TOPcloner™ TA Kit (Enzynomics, Daejeon, Korea) and the DNA sequences of the cloned PCR products were determined by Bioneer Sequencing Service (Daejeon, Korea). For accuracy, the DNA barcode analysis process was performed independently thrice from the genome DNA preparation stage.

### 4.5. Analysis of DNA Sequences and Preparation of the Dendrogram

DNA sequences were analyzed using ClustalW multiple sequence alignment (BioEdit, v7.0.9; available from http://www.mbio.ncsu.edu/BioEdit/page2.html) and confirmed using multiple sequence alignment in the multiple alignment program for amino acid or nucleotide sequences (MAFFT, v7; available from https://mafft.cbrc.jp/alignment/server) [37]. To verify the polymorphisms, represented by International Union of Pure and Applied Chemistry (IUPAC) nucleotide codes in the sequence data, all sequences were generated at least twice. The chromatograms of nucleotide sequences, which were provided by the Bioneer Sequencing Service, were compared. Evolutionary analyses were conducted in MEGA X (v10.0.5; available from https://www.megasoftware.net/). Phylogenic trees were constructed for the ITS region by using MAFFT (multiple alignment, v7.407_1), BMGE (alignment curation, v.1.12_1) [38], PhyML (tree inference based on the maximum-likelihood, v.3.1_1) [39], and workflow (PhyML/OneClick, available from https://ngphylogeny.fr/) [40]. Phylogenetic analysis of the concatenated regions of five DNA barcodes (ITS and four plastids) was constructed using the PhyML+SMS/OneClick method, which is shown in the MAFFT, BMGE, and PhyML+SMS (maximum likelihood-based inference of phylogenetic trees with Smart Model Selection, available from https://ngphylogeny.fr/) [40] workflow. All the analyzed sequences were compared with those available in the NCBI GenBank database using BLAST [41]. Newly determined nucleotide sequences were deposited in the NCBI GenBank database. Unlike previous research, we used three other subfamilies of the Zingiberales as an outgroup (*Siphonochilus kirkii*: Siphonochiloideae; *Camptandra parvula*: Zingiberoideae; and *Tamijia flagellaris*: Tamijioideae) [42].

### 4.6. Preparation of Samples for HPLC Analysis

Dried Amomi Fructus samples were pulverized and homogenized through a testing sieve. A 100 mg of sample powder was weighed accurately and sonicated with 1 mL of solvent mixture (methanol:water = 1:1) using an ultrasonic extractor (Power Sonic 520; Hwashin Tech, Daegu, Korea) for 30 min. Then, the extract was centrifuged at 10,770× *g* for 5 min and filtered through a 0.2 μm syringe filter (BioFact; Daejeon, Korea) prior to HPLC analysis.

### 4.7. HPLC Conditions for Chromatographic Profiling

Chromatographic analysis was performed using an Agilent 1260 liquid chromatography system (Agilent Technologies; Palo Alto, CA, USA) equipped with an autosampler, degasser, solvent pump, and DAD. The data were processed using ChemStation (Agilent Technologies). The separation of compounds was conducted on a Capcell Pak Mg II C_18_ column (4.6 mm × 250 mm, 5 μm; Shiseido, Tokyo, Japan) at 35 °C. The flow rate was 1 mL/min and the injection volume was 10 μL. The mobile phase consisted of water containing 0.1% TFA (A) and acetonitrile (B), with the following gradient elution: 8% (B) over 0–5 min, 8–30% (B) over 5–30 min, 30% (B) over 30–32 min, 30–85% (B) over 32–55 min, 85% (B) over 55–57 min, and then re-equilibrated to 8% (B) until the end of the analysis. Detection was performed using a UV detector at wavelengths of 225, 265, 280, 290, 310, and 320 nm.

The precision of selected peaks was determined by analyzing their retention times and absolute areas thrice within a day (intraday precision) and over three consecutive days (interday precision). Precisions are represented as relative standard deviations (RSDs), where RSD (%) = ((standard deviation/mean value) × 100).

### 4.8. Chemometric Statistical Analysis

Forty-two samples that were genetically identified were recoded and the relevance between samples was determined using chemometric tools including principal component analysis, hierarchical clustering analysis, heatmap analysis, and Pearson’s correlation analysis. In total, 77 peaks were selected as profiling peaks (the area of each peak > 1.0% of the total peak area) at their optimal UV absorption, and their absolute areas were calculated by peak area integration for chromatographic fingerprinting. A matrix comprising of the rows (Amomi Fructus sample) and columns (absolute area of each profiling peak) was used to construct the principle component (PC) plot, dendrogram, heatmap, and to calculate the Pearson’s correlation coefficient. Tukey’s test was used to compare the absolute area of each peak among the samples of single *Amomum* species. Chemometric analyses and Tukey’s test were conducted using the open source software R (v. 4.0.2; The R Foundation for Statistical Computing).

## 5. Conclusions

In this study, forty-two Amomi Fructus from various locational origins were phylogenetically identified using ITS and chloroplast genome-based DNA barcoding analysis and thereafter, their chromatographic profiles were compared using chemometric and correlation analysis for chemotaxonomic monitoring of Amomi Fructus. Eight *A. villosum* and six *A. longiligulare* samples showed closer genetic and chemical relations than the other non-medicinal *Amomum* adulterants. *A. ghaticum* samples represented higher chemical correlations with medicinally available *A. villosum* and six *A. longiligulare* samples than *A. microcarpum* samples did. Fifteen samples of *Amomum* species mixtures showed a variety of chemical relations with genuine *Amomum* species and their adulterants with four samples having zero or negative correlations. Chemotaxonomic monitoring using chromatographic profiling with chemometric analysis provides species-specific classification of Amomi Fructus in accordance with their phylogenetic relations.

## Figures and Tables

**Table 1:** Amomi Fructus samples identified using DNA barcode analysis of the internal transcribed spacer (ITS) region.

Code	Geographic Origin	Sample Type	Species Identification	Re-Code
AF01	China	F	*Amomum longiligulare*	ALS01
AF02	China	F	*A. ghaticum*	AGS01
AF03	China	F	*A. longiligulare*	ALS02
AF11	Myanmar	S.P.	*A. ghaticum*	AGS02
AF12	China	F	*A. villosum*	AVS01
AF14	Myanmar	F	*A. ghaticum*	AGS03
AF15	Vietnam	S	*A. microcarpum/A. longiligulare*	SM01
AF16	Vietnam	S	*A. microcarpum/A. longiligulare*	SM02
AF17	Myanmar	S	*A. ghaticum*	AGS04
AF21	China	F	*A. longiligulare*	ALS03
AF23	Myanmar	S	*A. ghaticum*	AGS05
AF24	Myanmar	S	*A. longiligulare/A. ghaticum*	SM03
AF25	Myanmar	S	*A. microcarpum*	AMS01
AF26	Myanmar	S	*A. ghaticum*	AGS06
AF27	Myanmar	S.P.	*A. ghaticum/A. uliginosum*	SM04
AF28	Myanmar	S	*A. longiligulare*	ALS04
AF29	China	S	*A. villosum*	AVS02
AF30	Myanmar	S	*A. microcarpum*	AMS02
AF31	Myanmar	S	*A. ghaticum*	AGS07
AF32	Myanmar	S	*A. ghaticum*	AGS08
AF33	Myanmar	S	*A. microcarpum*	AMS03
AF34	Myanmar	S	*A. ghaticum*	AGS09
AF35	Vietnam	P	*A. villosum/A. uliginosum/A. villosum* var. *villosum*	SM05
AF41	Vietnam	F	*A. longiligulare/A. villosum*	SM06
AF42	Vietnam	F	*A. tomrey* var. *tomrey/A. koenigii*	SM07
AF43	Vietnam	F	*A. longiligulare/A. villosum*	SM08
AF44	Vietnam	F	*A. koenigii/A. tomrey* var. *tomrey/A. chinense*	SM09
AF45	Vietnam	F	*A. longiligulare/A. villosum*	SM10
AF46	Vietnam	F	*A. koenigii/A. echinocarpum/A. chinense*	SM11
AF47	Vietnam	F	*A. longiligulare/A. villosum*	SM12
AF48	Vietnam	F	*A. koenigii/A. echinocarpum/A. chinense*	SM13
AF50	China	F	*A. villosum*	AVS07
AF51	China	F	*A. villosum*	AVS08
AF52	China	F	*A. longiligulare*	ALS05
AF53	China	F	*A. villosum*	AVS03
AF54	China	F	*A. villosum*	AVS04
AF55	Myanmar	S.P.	*A. longiligulare/A. microcarpum/A. villosum*	SM14
AF56	China	F	*A. villosum*	AVS05
AF57	China	F	*A. villosum*	AVS06
AF58	China	F	*A. longiligulare*	ALS06
AF59	Vietnam	S	*A. villosum/A. uliginosum/A. villosum* var. *villosum*	SM15
AF60	China	S	*A. microcarpum*	AMS04

F: whole fruits with pericarp; S: whole seeds without pericarp; S.P.: crushed seeds without pericarp; P: finely grinded seeds without pericarp.

**Table 2:** List of reference samples compared with Amomi Fructus and its adulterants.

No.	Accession Code	Scientific Name	Medicinal Name
1	AV01	*Amomum villosum* Lour. (= *Wurfbainia villosa* (Lour.) Skornick. and A.D. Poulsen)	Amomi Fructus ^a^
2	AV02		
3	AV03		
4	AL01	*Amomum longiligulare* T.L. Wu (= *Wurfbainia longiligularis* (T.L. Wu) Skornick. and A.D. Poulsen)	Amomi Fructus ^b^
5	AL02		
6	AL03		
7	AK01	*Amomum verum* Blackw. (= *Amomum krervanh* Pierre ex Gagnep.)	Amomi Fructus Rotundus
8	AK02		
9	AC01	*Amomum compactum* Sol. ex Maton	
10	AC02		
11	ATK01	*Amomum tsao-ko* Crevost and Lemarié (= *Amomum tsaoko*)	*Amomi tsao-ko* Fructus
12	ATK02		
13	AH01	*Alpinia hainanensis* K. Schum. (= *Alpinia katsumadae* Hayata)	*Alpiniae katsumadai* Semen
14	AH02		
15	AO01	*Alpinia oxyphylla* Miq.	*Alpiniae oxyphyllae* Fructus
16	AO02		
17	AOR01	*Alpinia officinarum* Hanc	*Alpiniae officinari* Rhizoma
18	AOR02		
19	ACC01	*Alpinia conchigera*	jie bian shan jiang ^c^
20	ACC02		
21	AZ01	*Alpinia zerumbet*	yan shan jiang ^c^
22	AZ02		
23	AM01	*Alpinia malaccensis*(N.L.Burman) Roscoe	mao ban shan jiang ^c^
24	AG01	*Alpinia galanga* (L.) Willd.	Galangae Fructus
25	EC01	*Elettaria cardamomum* (L.) Maton (= *Amomum cardamomum* L., *Alpinia cardamomum* (L.) Roxb.)	Cardamomi Fructus
26	EC02		

^a^ The Korean Pharmacopoeia, 11th edition, ^b^ Pharmacopoeia of the Peoples Republic of China and Taiwan Herbal Pharmacopeia, ^c^ Flora of China.

**Table 3:** Amplicon size of plastid loci and nuclear barcode region in Amomi Fructus and adulterants species and the sequence characteristics (single and in different multilocus combination).

Barcode Target	Amplicon Size (bp)	Aligned Length (bp)	Conserved Sites	Variable Sites	Parsimony Informative Sites	Singleton Site
**ITS**	**670**	**645–665**	**492**	**185**	**164**	21
*matk*	940	933	885	48	48	none
*rbcL*	750	743	657	86	12	74
*psbA-trnH*	830	800–836	820	49	35	14
*trnL-F* intergenic spacer	420	395–415	368	55	19	36
*matk* + *rbcL*	-	1676	1542	134	60	74
*psbA-trnH* + *trnL-F* intergenic spacer	-	1195-1251	1188	104	54	50
*mark* + *rbcL* + *psbA-trnH*	-	2479–2512	2362	183	95	88
*mark* + *rbcL* + *trnL-F* intergenic spacer	-	2071–2091	1910	189	79	110
Four plastid targets	-	2874–2927	2730	238	114	124

**Table 4:** Summary of Pearson’s correlation coefficients of Amomi Fructus samples.

Sample	Value	AVS	ALS	AGS	AMS	SM
AVS	Mean	0.785				
	Median	0.888				
	Max	0.987				
	Min	0.303				
ALS	Mean	0.829	0.895			
	Median	0.870	0.934			
	Max	0.995	0.988			
	Min	0.385	0.769			
AGS	Mean	0.767	0.883	0.980		
	Median	0.802	0.911	0.982		
	Max	0.949	0.963	0.995		
	Min	0.436	0.680	0.950		
AMS	Mean	0.449	0.352	0.199	0.802	
	Median	0.514	0.323	0.197	0.827	
	Max	0.711	0.645	0.271	0.958	
	Min	0.059	0.187	0.154	0.631	
SM	Mean	0.427	0.466	0.474	0.267	0.306
	Median	0.474	0.464	0.507	0.180	0.091
	Max	0.959	0.985	0.992	0.874	0.994
	Min	−0.049	−0.053	−0.048	−0.060	−0.065

## Supplementary Materials

The following are available online. Figure S1: phylogenetic tree; Figures S2–S3: chromatograms of Amomi Fructus; Figure S4: absolute area of profiling peaks; Figures S5–S9: average Pearson’s correlation coefficients. Table S1: identity matrix of DNA barcode regions; Table S2: retention times and detection wavelengths of profiling peaks; Table S3: precisions; Table S4: Pearson’s correlation coefficients.
