# Supplementary material for: Chemotaxonomic Monitoring of Genetically Authenticated Amomi Fructus Using High-Performance Liquid Chromatography–Diode Array Detector with Chemometric Analysis
Source: Molecules. 2020 Oct 7;25(19):4581. doi: 10.3390/molecules25194581 (PMC7583901; doi:10.3390/molecules25194581)
Supplement: Supplementary file 1 [file molecules-25-04581-s001.zip › Supplement Materials.docx]

Chemotaxonomic monitoring of genetically authenticated Amomi Fructus using high–performance liquid chromatography with chemometric analysis

Eui-Jeong Doh^1,‡^, Guemsan Lee^2,‡^, Hyun-Jong Jung^3^, Kang-Beom Kwon^4^ and Jung-Hoon Kim^5,*^

^1^Research Center of Traditional Korean Medicine, Wonkwang University, Iksan, Republic of Korea, 54538

^2^Department of Herbology, College of Korean Medicine, Wonkwang University, Iksan, Republic of Korea, 54538

^3^Department of Diagnostics, College of Korean Medicine, Wonkwang University, Iksan, Republic of Korea, 54538

^4^Department of Korean Medicinal Physiology, College of Korean Medicine, Wonkwang University, Iksan, Republic of Korea, 54538

^5^Division of Pharmacology, School of Korean Medicine, Pusan National University, Yangsan, Republic of Korea, 50612

^*^Correspondence: kmsct@pusan.ac.kr; Tel: +82-51-510-8456

^‡^These authors contributed equally to this work.


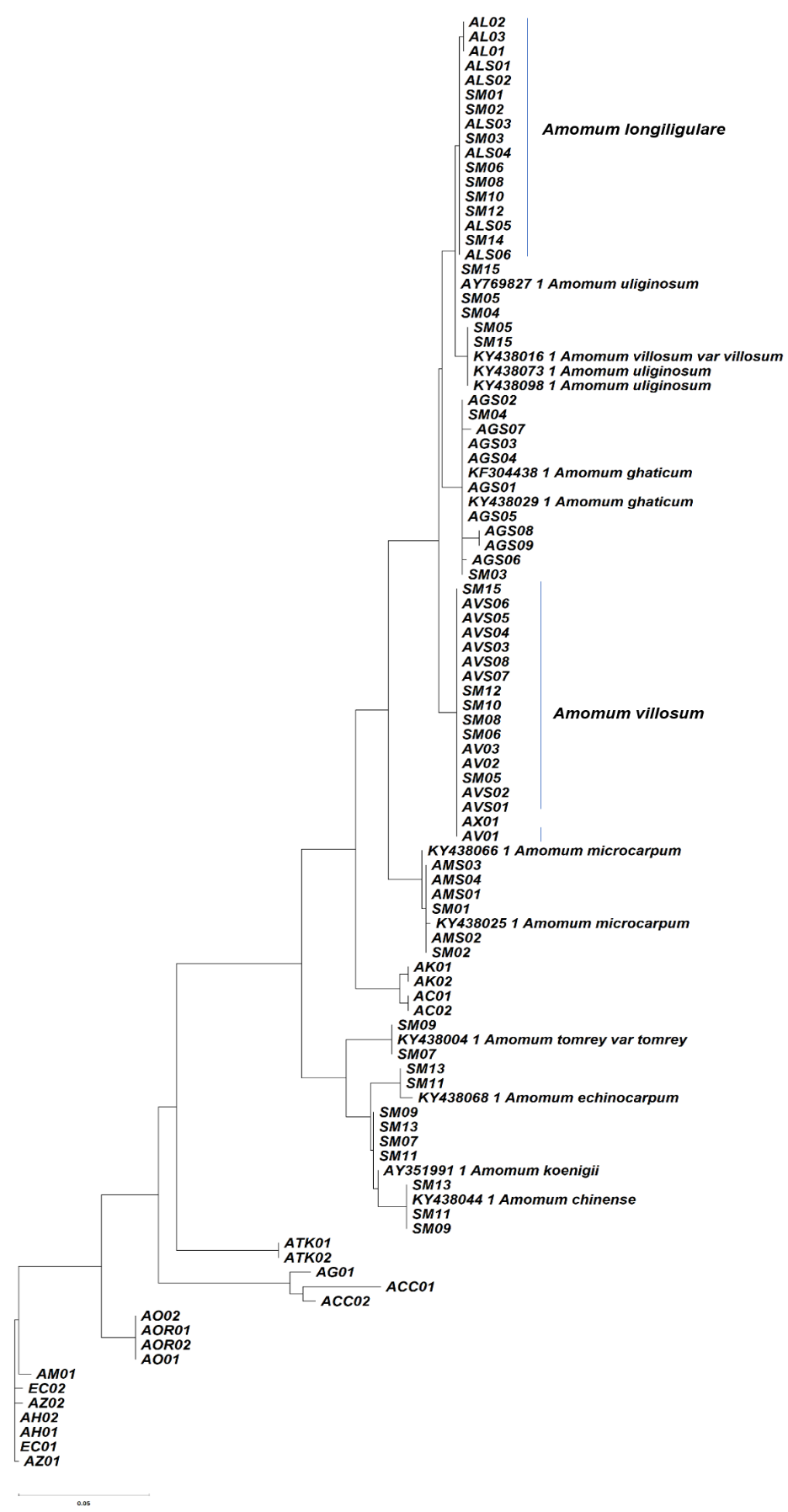


Figure S1. Maximum likelihood-based inference of phylogenetic tree based on nucleotide sequences of ITS region. AX01: *Amomum villosum* var. *xanthioides* nucleotide sequence of KJ151892.


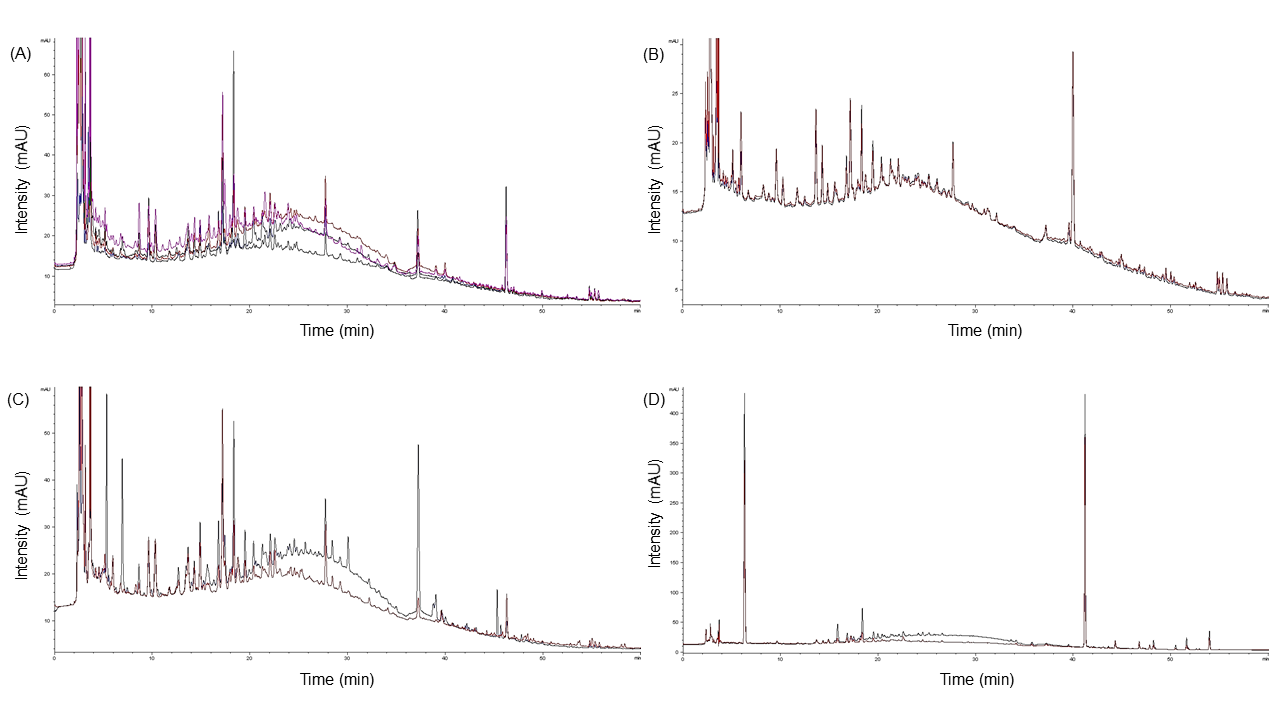


Figure S2. Overlapped chromatograms of mixed species of Amomi Fructus samples (more than two samples) at 280 nm of detection wavelength (DAD). A, *Amomum villosum* + *A. longiligulare* samples (SM06, -08, -10, and -12); B, *A. longiligulare* + *A. microcarpum* samples (SM01 and -02); C, *A. villosum* + *A. uliginosum* + *A. villosum* var. *villosum* samples (SM05 and -15); D, *A. koenigii* + *A. echinocarpum* + *A. chinense* samples (SM11 and -13).


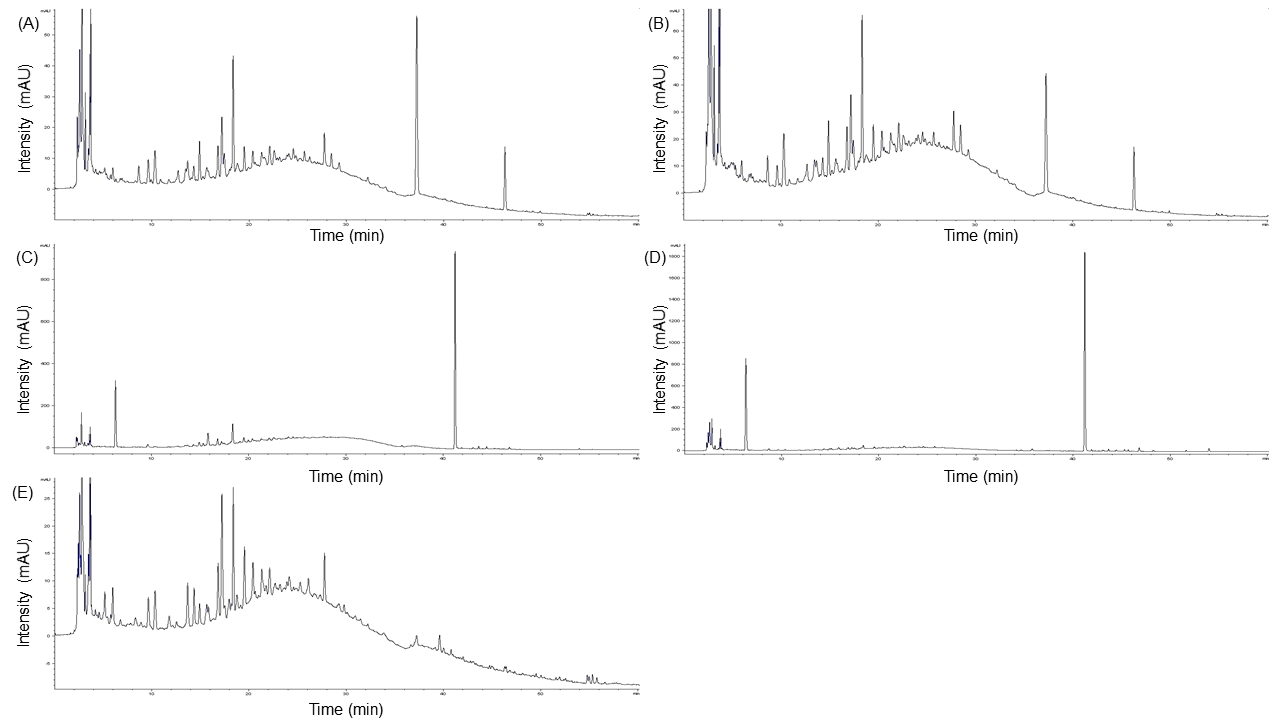


Figure S3. Chromatograms of mixed species of Amomi Fructus samples (one sample) at 280 nm of detection wavelength (DAD). A, *Amomum longiligulare* + *A. ghaticum* sample (SM03); B, *A. ghaticum* + *A. uliginosum* sample (SM04); C, *A. tomrey* var. *tomrey* + *A. koenigii* sample (SM07); D, *A. koenigii* + *A. echinocarpum* + *A. chinense* sample (SM09); E, *A. longiligulare* + *A. microcarpum* + *A. villosum* sample (SM14).


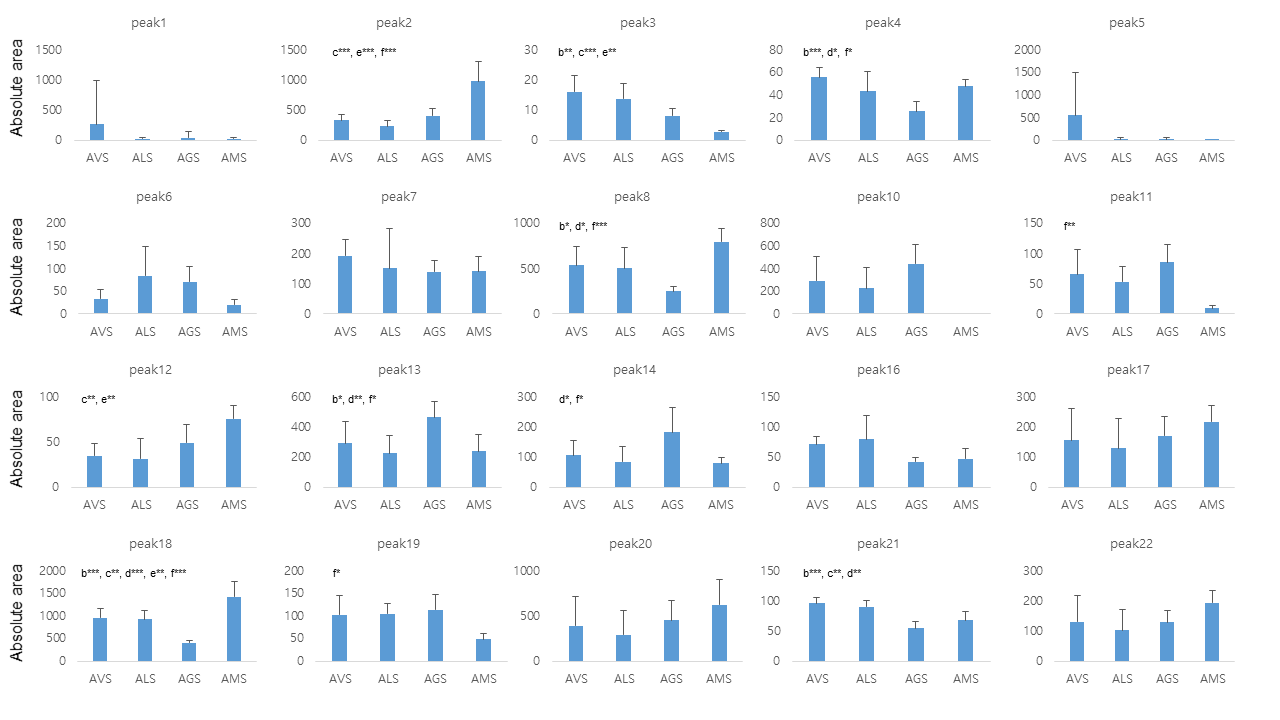

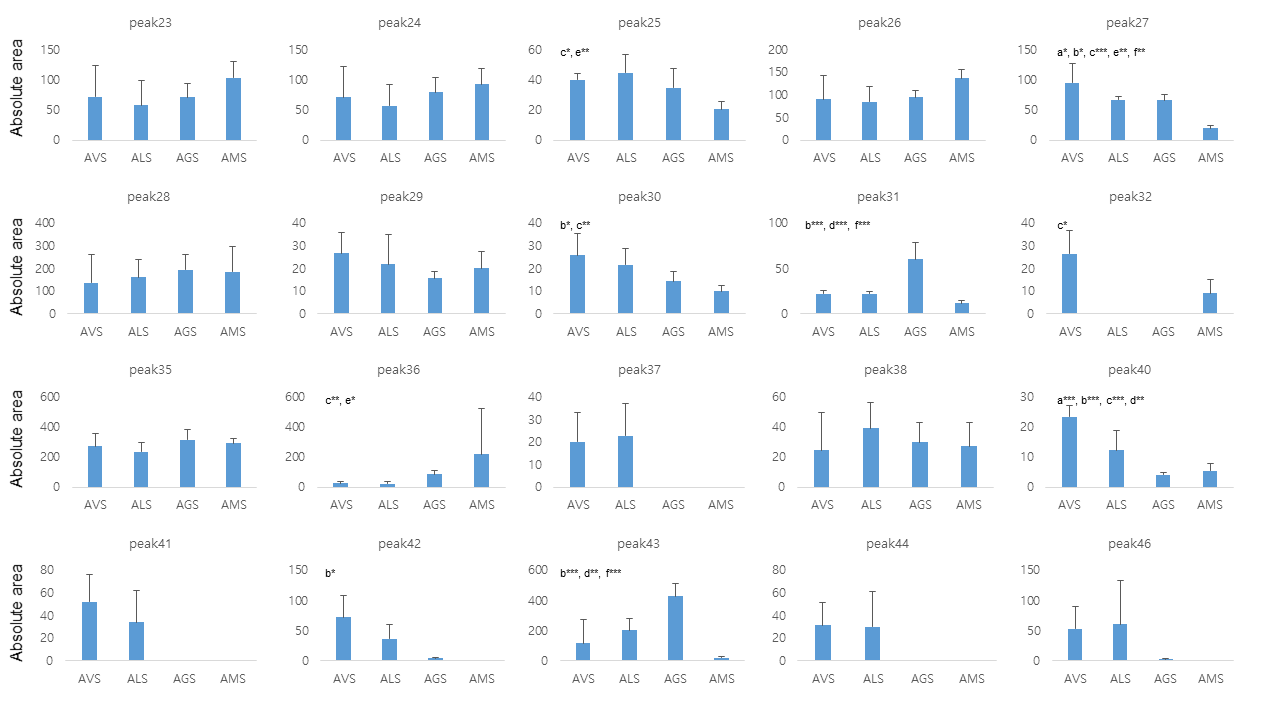

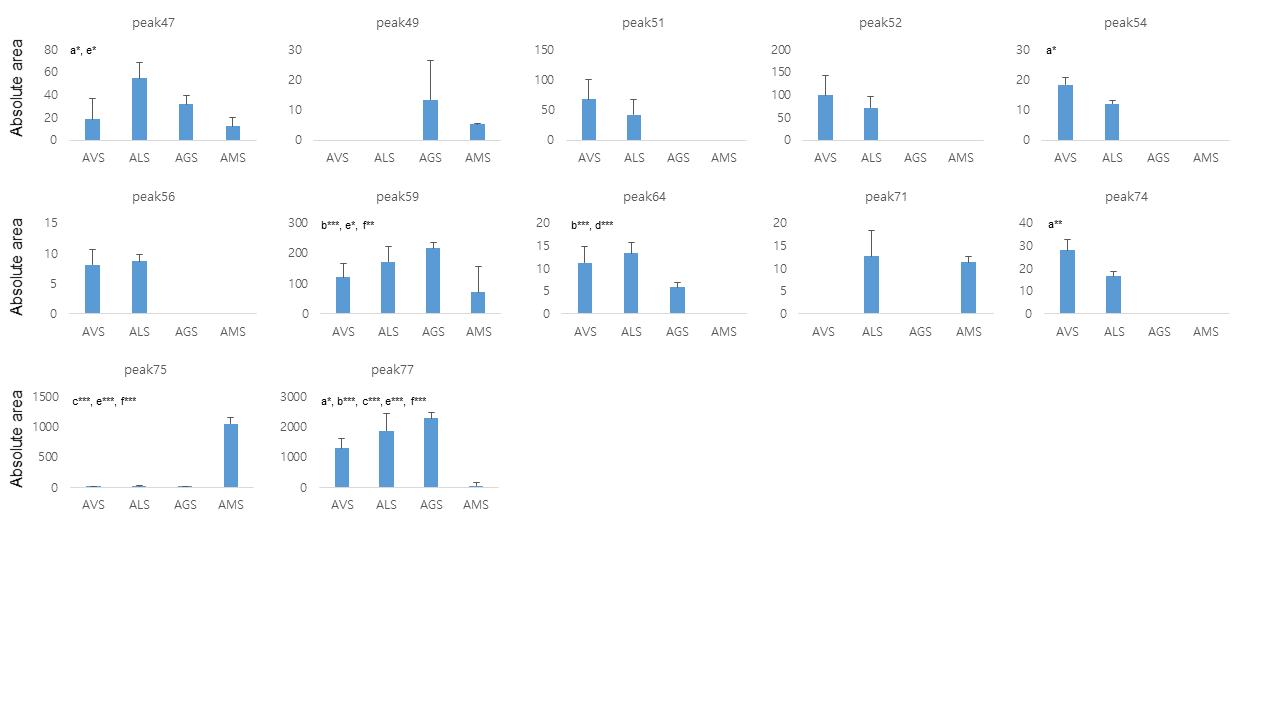


Figure S4. Absolute areas of profiling peaks from the samples of four single *Amomum* species. AVS, *A. villosum* samples; ALS, *A. longiligulare* samples AGS, *A. ghaticum* samples AMS, *A. microcarpum* samples.

Difference in peaks areas among AVS and ALS (a), AVS and AGS (b), AVS and AMS (c), ALS and AGS (d), ALS and AMS (e), AGS and AMS (f) when compared using the Tukey’s test, with a significance at ^*^*p* < 0.05, ^**^*p* < 0.01, and ^***^*p* < 0.001.


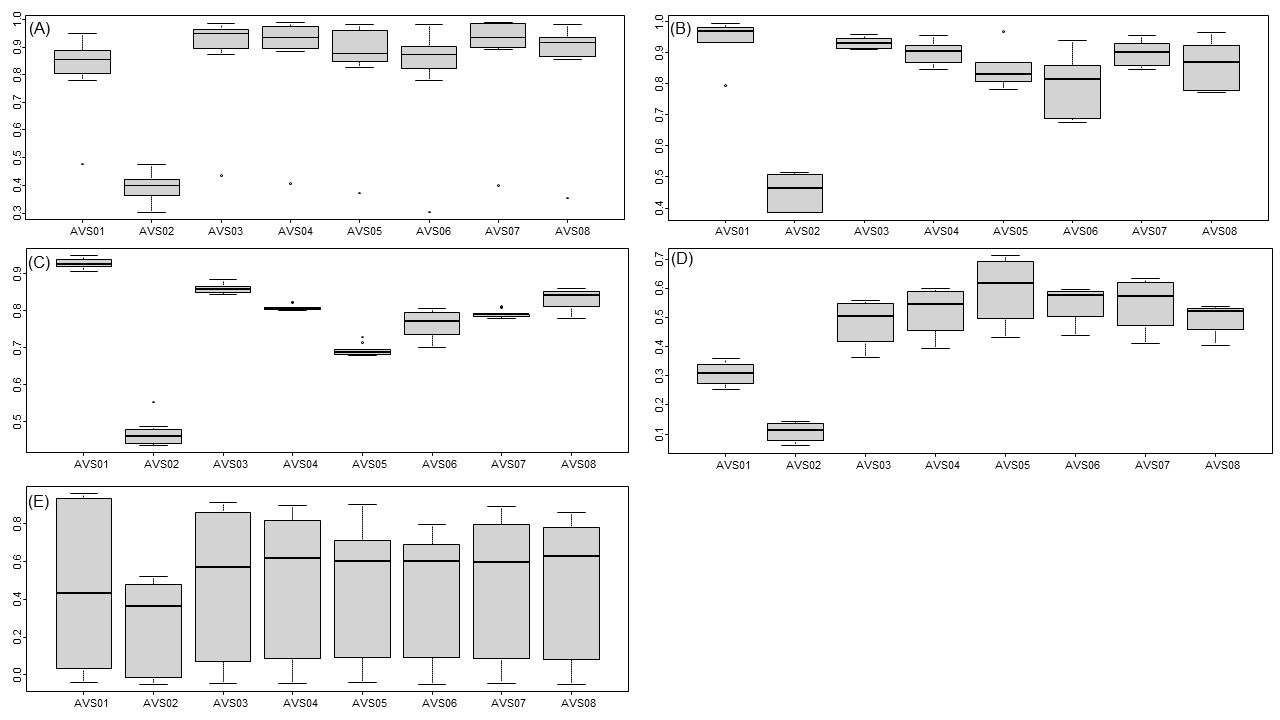


Figure S5. Average Pearson’s correlation coefficients of *Amomum villosum* samples (AVS) to other Amomi Fructus samples with AVS (A), ALS (B), AGS (C), AMS (D), and SM (E).


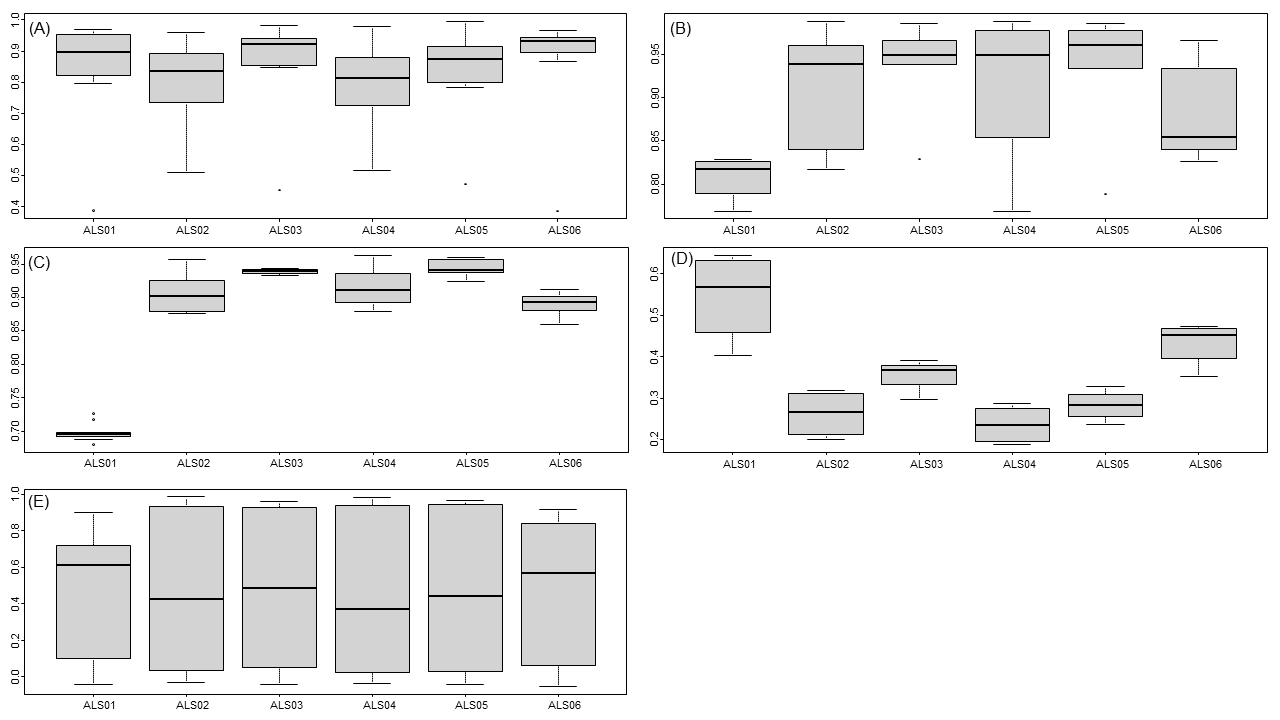


Figure S6. Average Pearson’s correlation coefficients of *Amomum longiligulare* samples (ALS) to other Amomi Fructus samples with AVS (A), ALS (B), AGS (C), AMS (D), and SM (E).


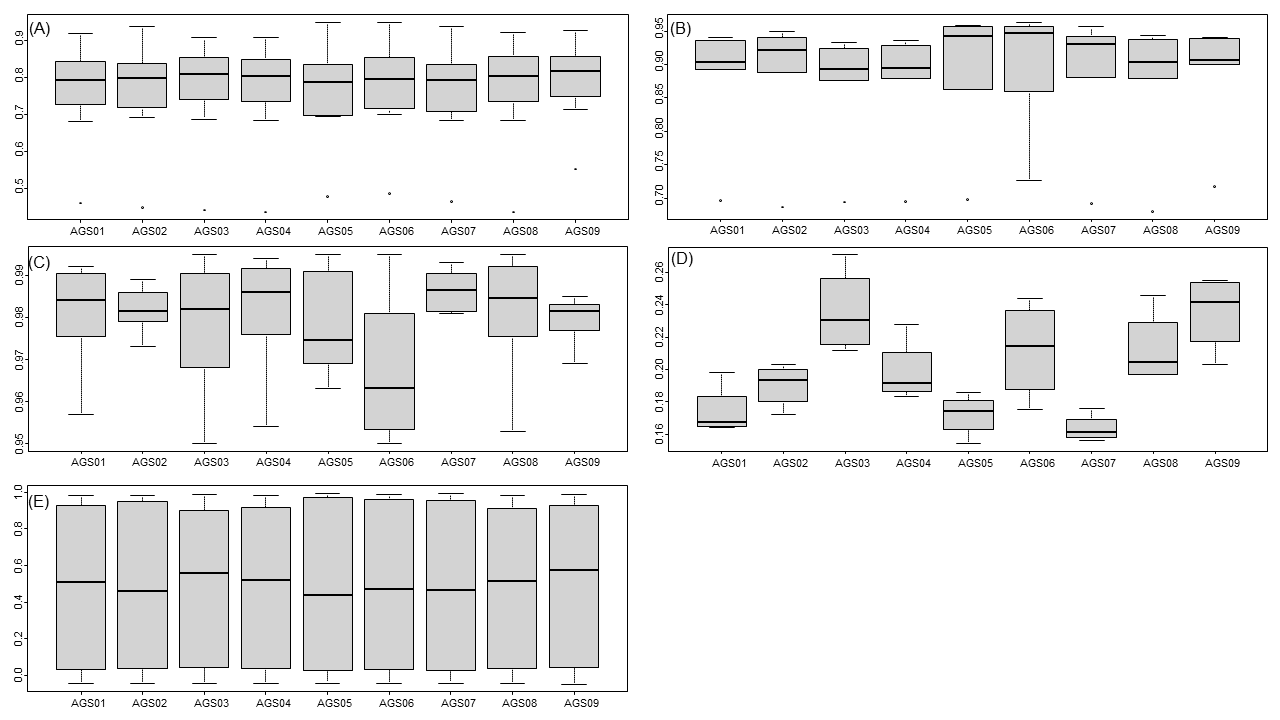


Figure S7. Average Pearson’s correlation coefficients of *Amomum ghaticum* samples (AGS) to other Amomi Fructus samples with AVS (A), ALS (B), AGS (C), AMS (D), and SM (E).


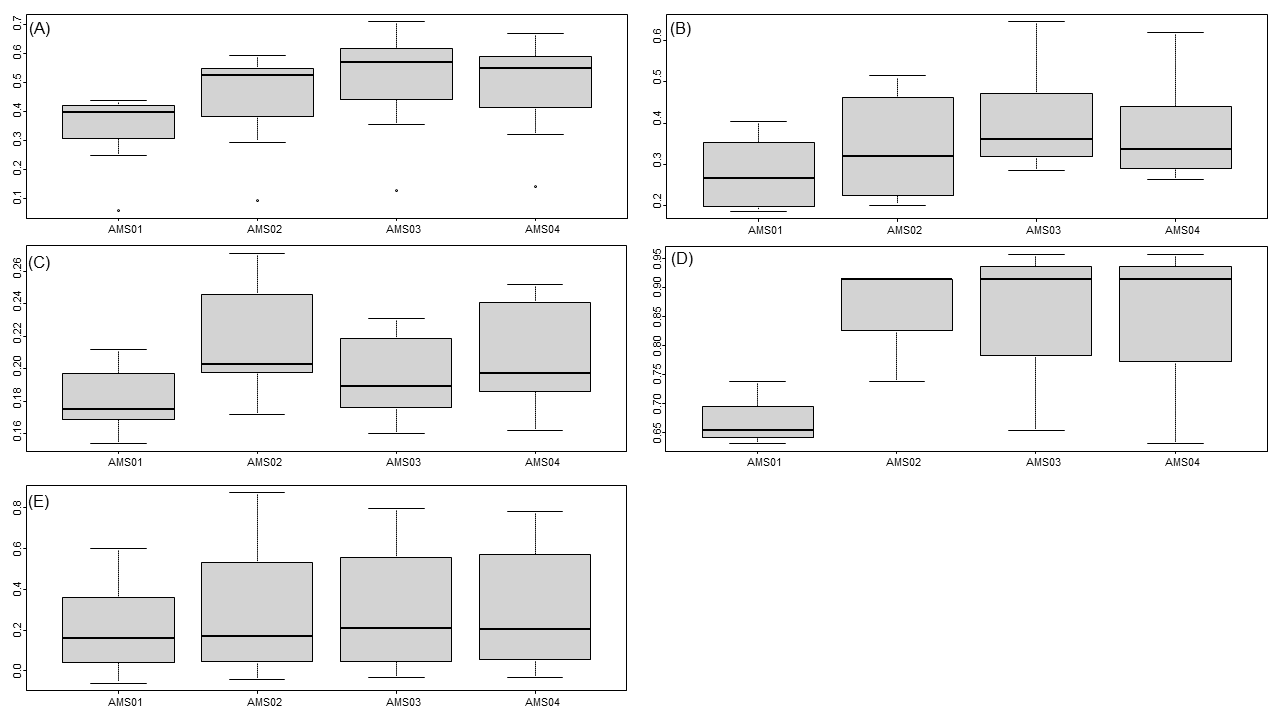


Figure S8. Average Pearson’s correlation coefficients of *Amomum microcarpum* samples (AMS) to other Amomi Fructus samples with AVS (A), ALS (B), AGS (C), AMS (D), and SM (E).

Figure S9. Average Pearson’s correlation coefficients of *Amomum* species mixed samples (SM) to other Amomi Fructus samples with AVS (A), ALS (B), AGS (C), AMS (D), and SM (E).


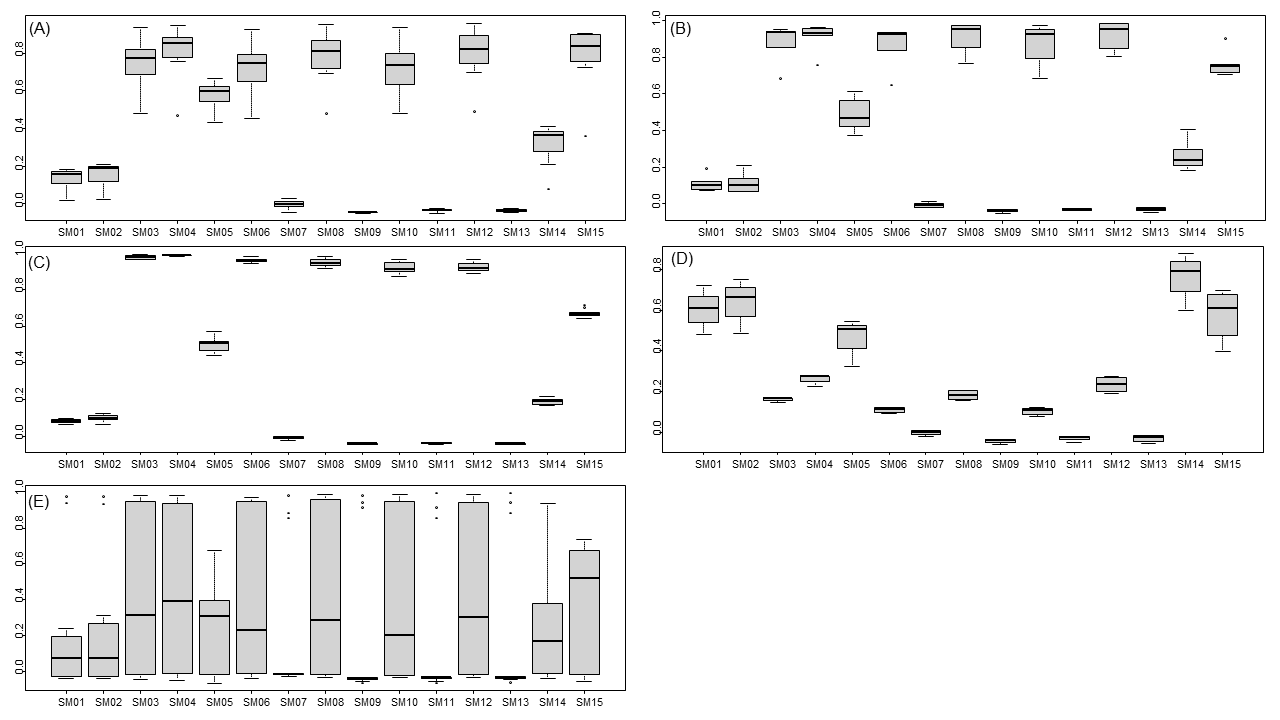


Table S1. Sequence Identity Matrix of five DNA barcode regions (excel file attached).

Table S2. Retention times and detection wavelengths of profiling peaks in Amomi Fructus samples

| Peak No. | Retention time (min) | Detection wavelength (nm) |  | Peak No. | Retention time (min) | Detection wavelength (nm) |
| --- | --- | --- | --- | --- | --- | --- |
| peak1 | 5.313 | 310 |  | peak40 | 32.191 | 310 |
| peak2 | 5.958 | 225 |  | peak41 | 33.292 | 310 |
| peak3 | 6.291 | 290 |  | peak42 | 33.603 | 320 |
| peak4 | 6.687 | 265 |  | peak43 | 37.217 | 280 |
| peak5 | 6.928 | 280 |  | peak44 | 39.38 | 310 |
| peak6 | 8.609 | 310 |  | peak45 | 39.565 | 310 |
| peak7 | 9.561 | 265 |  | peak46 | 39.709 | 320 |
| peak8 | 10.246 | 265 |  | peak47 | 39.991 | 290 |
| peak9 | 10.42 | 320 |  | peak48 | 40.447 | 290 |
| peak10 | 12.611 | 225 |  | peak49 | 40.759 | 320 |
| peak11 | 13.408 | 280 |  | peak50 | 41.185 | 280 |
| peak12 | 13.631 | 280 |  | peak51 | 41.397 | 310 |
| peak13 | 14.236 | 225 |  | peak52 | 41.702 | 320 |
| peak14 | 14.83 | 280 |  | peak53 | 43.134 | 320 |
| peak15 | 15.304 | 320 |  | peak54 | 43.503 | 310 |
| peak16 | 15.742 | 280 |  | peak55 | 44.308 | 320 |
| peak17 | 16.726 | 280 |  | peak56 | 44.709 | 310 |
| peak18 | 17.144 | 265 |  | peak57 | 45.285 | 225 |
| peak19 | 17.392 | 280 |  | peak58 | 45.657 | 265 |
| peak20 | 18.293 | 280 |  | peak59 | 46.297 | 290 |
| peak21 | 18.739 | 290 |  | peak60 | 46.687 | 320 |
| peak22 | 19.435 | 280 |  | peak61 | 46.938 | 290 |
| peak23 | 20.316 | 280 |  | peak62 | 47.781 | 320 |
| peak24 | 21.232 | 280 |  | peak63 | 48.228 | 320 |
| peak25 | 21.527 | 310 |  | peak64 | 49.891 | 310 |
| peak26 | 22.076 | 280 |  | peak65 | 50.488 | 320 |
| peak27 | 22.522 | 310 |  | peak66 | 51.619 | 265 |
| peak28 | 22.607 | 225 |  | peak67 | 52.102 | 290 |
| peak29 | 22.828 | 310 |  | peak68 | 52.632 | 320 |
| peak30 | 23.869 | 310 |  | peak69 | 53.223 | 290 |
| peak31 | 24.505 | 310 |  | peak70 | 53.949 | 320 |
| peak32 | 24.865 | 280 |  | peak71 | 54.798 | 225 |
| peak33 | 25.646 | 290 |  | peak72 | 55.096 | 320 |
| peak34 | 26.551 | 310 |  | peak73 | 55.816 | 225 |
| peak35 | 27.679 | 265 |  | peak74 | 56.777 | 225 |
| peak36 | 28.42 | 310 |  | peak75 | 57.693 | 225 |
| peak37 | 28.778 | 310 |  | peak76 | 58.328 | 225 |
| peak38 | 29.236 | 320 |  | peak77 | 59.038 | 225 |
| peak39 | 30.032 | 280 |  |  |  |  |

Peak 18, vanillic acid; peak 35, Quercitrin.

Table S3. Intra- and interday precisions of profiling peaks in Amomi Fructus sample (ALS01)

| Peak No. | Intraday precision (*n* = 3) | | | | |  | Interday precision (*n* = 3 × 3) | | | | |
| --- | --- | --- | --- | --- | --- | --- | --- | --- | --- | --- | --- |
|  | Retention time (min) | |  | Absolute area | |  | Retention time (min) | |  | Absolute area | |
|  | AVR | RSD (%) |  | AVR | RSD (%) |  | AVR | RSD (%) |  | AVR | RSD (%) |
| 1 | 5.319 | 0.038 |  | 16.567 | 3.432 |  | 5.319 | 0.118 |  | 16.500 | 2.794 |
| 2 | 5.968 | 0.067 |  | 239.600 | 2.643 |  | 5.969 | 0.166 |  | 240.667 | 2.269 |
| 3 | 6.278 | 0.066 |  | 6.967 | 5.801 |  | 6.278 | 0.111 |  | 6.844 | 5.014 |
| 4 | 6.683 | 0.100 |  | 74.767 | 1.766 |  | 6.694 | 0.295 |  | 74.367 | 2.809 |
| 6 | 8.602 | 0.071 |  | 69.133 | 1.390 |  | 8.607 | 0.172 |  | 70.000 | 2.209 |
| 7 | 9.560 | 0.079 |  | 415.733 | 2.706 |  | 9.573 | 0.219 |  | 417.689 | 2.126 |
| 8 | 10.233 | 0.113 |  | 318.900 | 1.549 |  | 10.246 | 0.276 |  | 324.244 | 1.998 |
| 10 | 12.605 | 0.071 |  | 250.733 | 4.222 |  | 12.622 | 0.239 |  | 235.444 | 6.520 |
| 11 | 13.401 | 0.056 |  | 52.700 | 1.830 |  | 13.412 | 0.185 |  | 50.922 | 3.944 |
| 12 | 13.627 | 0.040 |  | 73.633 | 6.248 |  | 13.634 | 0.136 |  | 74.067 | 4.308 |
| 13 | 14.238 | 0.053 |  | 338.200 | 2.703 |  | 14.251 | 0.184 |  | 327.811 | 3.195 |
| 14 | 14.826 | 0.045 |  | 63.900 | 2.932 |  | 14.840 | 0.172 |  | 62.511 | 3.022 |
| 16 | 15.732 | 0.037 |  | 117.600 | 3.208 |  | 15.745 | 0.159 |  | 115.033 | 2.856 |
| 17 | 16.721 | 0.043 |  | 122.067 | 4.304 |  | 16.735 | 0.156 |  | 119.167 | 3.200 |
| 18 | 17.135 | 0.039 |  | 1275.567 | 1.128 |  | 17.145 | 0.123 |  | 1273.011 | 1.227 |
| 19 | 17.381 | 0.040 |  | 79.700 | 2.144 |  | 17.392 | 0.137 |  | 78.511 | 2.317 |
| 20 | 18.285 | 0.052 |  | 175.467 | 4.820 |  | 18.297 | 0.132 |  | 173.156 | 3.677 |
| 22 | 19.420 | 0.061 |  | 105.600 | 4.855 |  | 19.436 | 0.133 |  | 101.756 | 4.477 |
| 23 | 20.302 | 0.053 |  | 59.833 | 1.553 |  | 20.318 | 0.122 |  | 57.856 | 2.889 |
| 24 | 21.218 | 0.047 |  | 91.600 | 5.866 |  | 21.122 | 1.626 |  | 85.500 | 6.713 |
| 25 | 21.502 | 0.026 |  | 36.267 | 2.312 |  | 21.505 | 0.075 |  | 35.278 | 3.293 |
| 26 | 22.067 | 0.052 |  | 90.467 | 6.149 |  | 22.075 | 0.077 |  | 89.256 | 4.136 |
| 27 | 22.487 | 0.043 |  | 78.567 | 0.389 |  | 22.501 | 0.096 |  | 77.000 | 3.352 |
| 28 | 22.593 | 0.058 |  | 237.400 | 5.943 |  | 22.610 | 0.104 |  | 226.289 | 5.777 |
| 29 | 22.822 | 0.046 |  | 16.567 | 1.940 |  | 22.829 | 0.078 |  | 15.733 | 4.627 |
| 30 | 23.872 | 0.048 |  | 17.100 | 4.217 |  | 23.881 | 0.077 |  | 17.700 | 4.422 |
| 31 | 24.480 | 0.043 |  | 26.167 | 0.584 |  | 24.492 | 0.083 |  | 25.078 | 4.448 |
| 32 | 24.846 | 0.064 |  | 40.433 | 3.092 |  | 24.862 | 0.094 |  | 41.900 | 8.935 |
| 35 | 27.659 | 0.044 |  | 230.400 | 1.165 |  | 27.674 | 0.074 |  | 227.978 | 2.457 |
| 36 | 28.426 | 0.023 |  | 13.467 | 4.537 |  | 28.428 | 0.036 |  | 14.100 | 8.533 |
| 37 | 28.757 | 0.038 |  | 19.367 | 7.734 |  | 28.775 | 0.068 |  | 19.511 | 6.690 |
| 38 | 29.213 | 0.027 |  | 39.867 | 8.032 |  | 29.217 | 0.031 |  | 40.744 | 7.575 |
| 40 | 32.175 | 0.037 |  | 17.933 | 6.014 |  | 32.193 | 0.058 |  | 17.922 | 5.936 |
| 41 | 33.264 | 0.033 |  | 46.967 | 6.216 |  | 33.287 | 0.066 |  | 47.189 | 5.023 |
| 42 | 33.572 | 0.033 |  | 52.933 | 5.391 |  | 33.594 | 0.062 |  | 52.911 | 4.169 |
| 43 | 37.193 | 0.027 |  | 22.800 | 2.010 |  | 37.204 | 0.058 |  | 24.544 | 7.832 |
| 44 | 39.371 | 0.018 |  | 23.200 | 2.692 |  | 39.384 | 0.040 |  | 23.867 | 6.558 |
| 46 | 39.700 | 0.017 |  | 26.600 | 4.620 |  | 39.711 | 0.038 |  | 27.344 | 4.866 |
| 47 | 39.978 | 0.019 |  | 62.267 | 2.034 |  | 39.985 | 0.034 |  | 61.322 | 2.569 |
| 51 | 41.394 | 0.013 |  | 61.100 | 5.940 |  | 41.404 | 0.033 |  | 61.456 | 5.884 |
| 52 | 41.698 | 0.016 |  | 74.267 | 6.159 |  | 41.707 | 0.031 |  | 74.356 | 5.747 |
| 54 | 43.471 | 0.016 |  | 12.800 | 6.250 |  | 43.481 | 0.027 |  | 12.733 | 5.903 |
| 56 | 44.694 | 0.015 |  | 10.033 | 2.508 |  | 44.699 | 0.019 |  | 9.900 | 2.901 |
| 59 | 46.284 | 0.012 |  | 86.500 | 1.667 |  | 46.285 | 0.013 |  | 86.456 | 2.716 |
| 64 | 49.893 | 0.014 |  | 14.067 | 6.605 |  | 49.900 | 0.015 |  | 14.400 | 5.663 |
| 74 | 56.760 | 0.019 |  | 22.733 | 5.500 |  | 56.765 | 0.013 |  | 22.989 | 6.294 |
| 77 | 58.998 | 0.012 |  | 991.500 | 1.364 |  | 59.002 | 0.011 |  | 980.622 | 1.923 |

Table S4. Pearson’s correlation coefficients of Amomi Fructus samples

|  | AVS01 | AVS02 | AVS03 | AVS04 | AVS05 | AVS06 | AVS07 | AVS08 | ALS01 | ALS02 | ALS03 | ALS04 | ALS05 | ALS06 |
| --- | --- | --- | --- | --- | --- | --- | --- | --- | --- | --- | --- | --- | --- | --- |
| AVS01 | 1.000 |  |  |  |  |  |  |  |  |  |  |  |  |  |
| AVS02 | 0.477 | 1.000 |  |  |  |  |  |  |  |  |  |  |  |  |
| AVS03 | 0.948 | 0.435 | 1.000 |  |  |  |  |  |  |  |  |  |  |  |
| AVS04 | 0.885 | 0.406 | 0.978 | 1.000 |  |  |  |  |  |  |  |  |  |  |
| AVS05 | 0.827 | 0.372 | 0.952 | 0.968 | 1.000 |  |  |  |  |  |  |  |  |  |
| AVS06 | 0.780 | 0.303 | 0.872 | 0.902 | 0.865 | 1.000 |  |  |  |  |  |  |  |  |
| AVS07 | 0.892 | 0.399 | 0.984 | 0.987 | 0.983 | 0.905 | 1.000 |  |  |  |  |  |  |  |
| AVS08 | 0.856 | 0.355 | 0.918 | 0.934 | 0.878 | 0.981 | 0.933 | 1.000 |  |  |  |  |  |  |
| ALS01 | 0.795 | 0.387 | 0.919 | 0.954 | 0.969 | 0.846 | 0.955 | 0.871 | 1.000 |  |  |  |  |  |
| ALS02 | 0.959 | 0.509 | 0.914 | 0.869 | 0.808 | 0.688 | 0.860 | 0.780 | 0.817 | 1.000 |  |  |  |  |
| ALS03 | 0.982 | 0.452 | 0.958 | 0.923 | 0.848 | 0.859 | 0.920 | 0.924 | 0.829 | 0.938 | 1.000 |  |  |  |
| ALS04 | 0.979 | 0.515 | 0.911 | 0.846 | 0.781 | 0.677 | 0.845 | 0.772 | 0.769 | 0.988 | 0.949 | 1.000 |  |  |
| ALS05 | 0.995 | 0.472 | 0.942 | 0.887 | 0.815 | 0.782 | 0.885 | 0.863 | 0.789 | 0.960 | 0.985 | 0.977 | 1.000 |  |
| ALS06 | 0.932 | 0.385 | 0.945 | 0.924 | 0.867 | 0.940 | 0.929 | 0.967 | 0.826 | 0.840 | 0.966 | 0.854 | 0.934 | 1.000 |
| AGS01 | 0.919 | 0.461 | 0.844 | 0.804 | 0.680 | 0.772 | 0.780 | 0.841 | 0.697 | 0.901 | 0.936 | 0.904 | 0.940 | 0.892 |
| AGS02 | 0.939 | 0.448 | 0.865 | 0.803 | 0.693 | 0.743 | 0.791 | 0.811 | 0.687 | 0.912 | 0.941 | 0.930 | 0.950 | 0.888 |
| AGS03 | 0.907 | 0.442 | 0.849 | 0.809 | 0.688 | 0.806 | 0.790 | 0.861 | 0.694 | 0.876 | 0.933 | 0.879 | 0.924 | 0.906 |
| AGS04 | 0.909 | 0.436 | 0.847 | 0.810 | 0.684 | 0.795 | 0.786 | 0.853 | 0.695 | 0.879 | 0.936 | 0.887 | 0.929 | 0.901 |
| AGS05 | 0.949 | 0.478 | 0.867 | 0.802 | 0.695 | 0.701 | 0.789 | 0.785 | 0.698 | 0.946 | 0.939 | 0.957 | 0.959 | 0.863 |
| AGS06 | 0.949 | 0.487 | 0.886 | 0.822 | 0.729 | 0.701 | 0.811 | 0.780 | 0.727 | 0.957 | 0.935 | 0.963 | 0.957 | 0.859 |
| AGS07 | 0.938 | 0.465 | 0.856 | 0.802 | 0.683 | 0.735 | 0.782 | 0.814 | 0.692 | 0.926 | 0.942 | 0.935 | 0.956 | 0.880 |
| AGS08 | 0.921 | 0.437 | 0.853 | 0.807 | 0.683 | 0.797 | 0.790 | 0.859 | 0.680 | 0.879 | 0.943 | 0.893 | 0.938 | 0.912 |
| AGS09 | 0.926 | 0.553 | 0.871 | 0.824 | 0.714 | 0.784 | 0.809 | 0.845 | 0.718 | 0.901 | 0.940 | 0.910 | 0.939 | 0.900 |
| AMS01 | 0.251 | 0.059 | 0.363 | 0.393 | 0.432 | 0.438 | 0.411 | 0.405 | 0.403 | 0.199 | 0.297 | 0.187 | 0.236 | 0.353 |
| AMS02 | 0.296 | 0.094 | 0.470 | 0.517 | 0.562 | 0.596 | 0.533 | 0.537 | 0.514 | 0.225 | 0.367 | 0.202 | 0.273 | 0.462 |
| AMS03 | 0.358 | 0.129 | 0.559 | 0.600 | 0.711 | 0.579 | 0.634 | 0.526 | 0.645 | 0.318 | 0.391 | 0.286 | 0.328 | 0.472 |
| AMS04 | 0.321 | 0.142 | 0.534 | 0.573 | 0.669 | 0.568 | 0.608 | 0.511 | 0.620 | 0.304 | 0.369 | 0.264 | 0.289 | 0.439 |
| SM01 | 0.081 | 0.017 | 0.156 | 0.178 | 0.183 | 0.159 | 0.168 | 0.138 | 0.192 | 0.094 | 0.102 | 0.070 | 0.074 | 0.121 |
| SM02 | 0.082 | 0.022 | 0.184 | 0.198 | 0.212 | 0.190 | 0.196 | 0.151 | 0.208 | 0.092 | 0.105 | 0.067 | 0.068 | 0.138 |
| SM03 | 0.936 | 0.482 | 0.851 | 0.788 | 0.678 | 0.690 | 0.771 | 0.776 | 0.683 | 0.939 | 0.927 | 0.943 | 0.950 | 0.851 |
| SM04 | 0.949 | 0.468 | 0.902 | 0.856 | 0.757 | 0.799 | 0.842 | 0.861 | 0.758 | 0.930 | 0.958 | 0.932 | 0.961 | 0.917 |
| SM05 | 0.429 | 0.523 | 0.568 | 0.619 | 0.599 | 0.666 | 0.595 | 0.628 | 0.610 | 0.423 | 0.487 | 0.370 | 0.442 | 0.565 |
| SM06 | 0.923 | 0.456 | 0.813 | 0.751 | 0.625 | 0.672 | 0.735 | 0.770 | 0.648 | 0.916 | 0.926 | 0.935 | 0.937 | 0.835 |
| SM07 | -0.016 | -0.043 | -0.010 | 0.000 | -0.004 | 0.026 | 0.000 | 0.024 | 0.011 | -0.020 | -0.002 | -0.024 | -0.013 | 0.001 |
| SM08 | 0.954 | 0.480 | 0.891 | 0.842 | 0.745 | 0.689 | 0.824 | 0.787 | 0.763 | 0.972 | 0.944 | 0.976 | 0.964 | 0.851 |
| SM09 | -0.040 | -0.044 | -0.046 | -0.046 | -0.042 | -0.048 | -0.043 | -0.048 | -0.040 | -0.032 | -0.043 | -0.033 | -0.039 | -0.053 |
| SM10 | 0.936 | 0.480 | 0.837 | 0.764 | 0.669 | 0.598 | 0.755 | 0.709 | 0.686 | 0.953 | 0.906 | 0.971 | 0.942 | 0.793 |
| SM11 | -0.036 | -0.049 | -0.038 | -0.032 | -0.035 | -0.024 | -0.033 | -0.024 | -0.030 | -0.031 | -0.030 | -0.034 | -0.035 | -0.038 |
| SM12 | 0.959 | 0.491 | 0.913 | 0.869 | 0.788 | 0.696 | 0.852 | 0.791 | 0.802 | 0.985 | 0.946 | 0.984 | 0.963 | 0.850 |
| SM13 | -0.036 | -0.044 | -0.036 | -0.031 | -0.026 | -0.038 | -0.030 | -0.038 | -0.021 | -0.023 | -0.036 | -0.028 | -0.036 | -0.049 |
| SM14 | 0.211 | 0.079 | 0.345 | 0.390 | 0.411 | 0.382 | 0.382 | 0.347 | 0.407 | 0.214 | 0.257 | 0.180 | 0.205 | 0.298 |
| SM15 | 0.725 | 0.361 | 0.875 | 0.900 | 0.903 | 0.781 | 0.893 | 0.792 | 0.903 | 0.746 | 0.756 | 0.705 | 0.719 | 0.759 |

|  | AGS01 | AGS02 | AGS03 | AGS04 | AGS05 | AGS06 | AGS07 | AGS08 | AGS09 | AMS01 | AMS02 | AMS03 | AMS04 | SM01 |
| --- | --- | --- | --- | --- | --- | --- | --- | --- | --- | --- | --- | --- | --- | --- |
| AVS01 |  |  |  |  |  |  |  |  |  |  |  |  |  |  |
| AVS02 |  |  |  |  |  |  |  |  |  |  |  |  |  |  |
| AVS03 |  |  |  |  |  |  |  |  |  |  |  |  |  |  |
| AVS04 |  |  |  |  |  |  |  |  |  |  |  |  |  |  |
| AVS05 |  |  |  |  |  |  |  |  |  |  |  |  |  |  |
| AVS06 |  |  |  |  |  |  |  |  |  |  |  |  |  |  |
| AVS07 |  |  |  |  |  |  |  |  |  |  |  |  |  |  |
| AVS08 |  |  |  |  |  |  |  |  |  |  |  |  |  |  |
| ALS01 |  |  |  |  |  |  |  |  |  |  |  |  |  |  |
| ALS02 |  |  |  |  |  |  |  |  |  |  |  |  |  |  |
| ALS03 |  |  |  |  |  |  |  |  |  |  |  |  |  |  |
| ALS04 |  |  |  |  |  |  |  |  |  |  |  |  |  |  |
| ALS05 |  |  |  |  |  |  |  |  |  |  |  |  |  |  |
| ALS06 |  |  |  |  |  |  |  |  |  |  |  |  |  |  |
| AGS01 | 1.000 |  |  |  |  |  |  |  |  |  |  |  |  |  |
| AGS02 | 0.977 | 1.000 |  |  |  |  |  |  |  |  |  |  |  |  |
| AGS03 | 0.989 | 0.973 | 1.000 |  |  |  |  |  |  |  |  |  |  |  |
| AGS04 | 0.991 | 0.983 | 0.992 | 1.000 |  |  |  |  |  |  |  |  |  |  |
| AGS05 | 0.974 | 0.989 | 0.963 | 0.969 | 1.000 |  |  |  |  |  |  |  |  |  |
| AGS06 | 0.957 | 0.981 | 0.950 | 0.954 | 0.995 | 1.000 |  |  |  |  |  |  |  |  |
| AGS07 | 0.992 | 0.989 | 0.981 | 0.987 | 0.993 | 0.981 | 1.000 |  |  |  |  |  |  |  |
| AGS08 | 0.990 | 0.982 | 0.995 | 0.994 | 0.969 | 0.953 | 0.986 | 1.000 |  |  |  |  |  |  |
| AGS09 | 0.979 | 0.981 | 0.983 | 0.985 | 0.975 | 0.969 | 0.982 | 0.983 | 1.000 |  |  |  |  |  |
| AMS01 | 0.169 | 0.172 | 0.212 | 0.190 | 0.154 | 0.175 | 0.156 | 0.197 | 0.203 | 1.000 |  |  |  |  |
| AMS02 | 0.198 | 0.203 | 0.271 | 0.228 | 0.172 | 0.200 | 0.176 | 0.246 | 0.255 | 0.739 | 1.000 |  |  |  |
| AMS03 | 0.165 | 0.189 | 0.219 | 0.183 | 0.176 | 0.229 | 0.160 | 0.197 | 0.231 | 0.653 | 0.915 | 1.000 |  |  |
| AMS04 | 0.164 | 0.197 | 0.241 | 0.193 | 0.186 | 0.244 | 0.162 | 0.212 | 0.252 | 0.631 | 0.915 | 0.958 | 1.000 |  |
| SM01 | 0.065 | 0.086 | 0.090 | 0.080 | 0.074 | 0.093 | 0.065 | 0.075 | 0.097 | 0.479 | 0.719 | 0.615 | 0.597 | 1.000 |
| SM02 | 0.066 | 0.110 | 0.114 | 0.097 | 0.090 | 0.121 | 0.071 | 0.093 | 0.126 | 0.487 | 0.749 | 0.646 | 0.671 | 0.975 |
| SM03 | 0.976 | 0.975 | 0.965 | 0.963 | 0.992 | 0.985 | 0.991 | 0.966 | 0.967 | 0.147 | 0.166 | 0.166 | 0.170 | 0.063 |
| SM04 | 0.984 | 0.983 | 0.986 | 0.984 | 0.983 | 0.980 | 0.988 | 0.984 | 0.986 | 0.227 | 0.275 | 0.274 | 0.278 | 0.097 |
| SM05 | 0.507 | 0.455 | 0.557 | 0.517 | 0.439 | 0.469 | 0.464 | 0.513 | 0.572 | 0.324 | 0.507 | 0.502 | 0.543 | 0.194 |
| SM06 | 0.961 | 0.966 | 0.941 | 0.955 | 0.977 | 0.956 | 0.978 | 0.953 | 0.948 | 0.125 | 0.125 | 0.095 | 0.103 | 0.048 |
| SM07 | -0.004 | -0.015 | -0.002 | -0.001 | -0.019 | -0.024 | -0.012 | -0.004 | -0.011 | -0.021 | 0.012 | 0.000 | 0.004 | -0.027 |
| SM08 | 0.939 | 0.958 | 0.911 | 0.932 | 0.977 | 0.975 | 0.965 | 0.925 | 0.939 | 0.157 | 0.165 | 0.208 | 0.201 | 0.085 |
| SM09 | -0.044 | -0.043 | -0.046 | -0.043 | -0.041 | -0.041 | -0.042 | -0.045 | -0.048 | -0.060 | -0.042 | -0.032 | -0.032 | -0.039 |
| SM10 | 0.909 | 0.938 | 0.872 | 0.896 | 0.965 | 0.958 | 0.945 | 0.892 | 0.906 | 0.099 | 0.079 | 0.121 | 0.112 | 0.047 |
| SM11 | -0.036 | -0.043 | -0.037 | -0.036 | -0.041 | -0.044 | -0.039 | -0.036 | -0.044 | -0.047 | -0.018 | -0.023 | -0.021 | -0.040 |
| SM12 | 0.914 | 0.932 | 0.886 | 0.902 | 0.958 | 0.962 | 0.941 | 0.896 | 0.916 | 0.190 | 0.212 | 0.275 | 0.260 | 0.094 |
| SM13 | -0.043 | -0.045 | -0.045 | -0.043 | -0.040 | -0.040 | -0.042 | -0.046 | -0.048 | -0.053 | -0.029 | -0.016 | -0.016 | -0.036 |
| SM14 | 0.171 | 0.185 | 0.210 | 0.195 | 0.169 | 0.199 | 0.164 | 0.188 | 0.215 | 0.599 | 0.874 | 0.799 | 0.783 | 0.938 |
| SM15 | 0.641 | 0.666 | 0.671 | 0.662 | 0.670 | 0.714 | 0.650 | 0.656 | 0.700 | 0.396 | 0.556 | 0.659 | 0.694 | 0.243 |

|  | SM02 | SM03 | SM04 | SM05 | SM06 | SM07 | SM08 | SM09 | SM10 | SM11 | SM12 | SM13 | SM14 | SM15 |
| --- | --- | --- | --- | --- | --- | --- | --- | --- | --- | --- | --- | --- | --- | --- |
| AVS01 |  |  |  |  |  |  |  |  |  |  |  |  |  |  |
| AVS02 |  |  |  |  |  |  |  |  |  |  |  |  |  |  |
| AVS03 |  |  |  |  |  |  |  |  |  |  |  |  |  |  |
| AVS04 |  |  |  |  |  |  |  |  |  |  |  |  |  |  |
| AVS05 |  |  |  |  |  |  |  |  |  |  |  |  |  |  |
| AVS06 |  |  |  |  |  |  |  |  |  |  |  |  |  |  |
| AVS07 |  |  |  |  |  |  |  |  |  |  |  |  |  |  |
| AVS08 |  |  |  |  |  |  |  |  |  |  |  |  |  |  |
| ALS01 |  |  |  |  |  |  |  |  |  |  |  |  |  |  |
| ALS02 |  |  |  |  |  |  |  |  |  |  |  |  |  |  |
| ALS03 |  |  |  |  |  |  |  |  |  |  |  |  |  |  |
| ALS04 |  |  |  |  |  |  |  |  |  |  |  |  |  |  |
| ALS05 |  |  |  |  |  |  |  |  |  |  |  |  |  |  |
| ALS06 |  |  |  |  |  |  |  |  |  |  |  |  |  |  |
| AGS01 |  |  |  |  |  |  |  |  |  |  |  |  |  |  |
| AGS02 |  |  |  |  |  |  |  |  |  |  |  |  |  |  |
| AGS03 |  |  |  |  |  |  |  |  |  |  |  |  |  |  |
| AGS04 |  |  |  |  |  |  |  |  |  |  |  |  |  |  |
| AGS05 |  |  |  |  |  |  |  |  |  |  |  |  |  |  |
| AGS06 |  |  |  |  |  |  |  |  |  |  |  |  |  |  |
| AGS07 |  |  |  |  |  |  |  |  |  |  |  |  |  |  |
| AGS08 |  |  |  |  |  |  |  |  |  |  |  |  |  |  |
| AGS09 |  |  |  |  |  |  |  |  |  |  |  |  |  |  |
| AMS01 |  |  |  |  |  |  |  |  |  |  |  |  |  |  |
| AMS02 |  |  |  |  |  |  |  |  |  |  |  |  |  |  |
| AMS03 |  |  |  |  |  |  |  |  |  |  |  |  |  |  |
| AMS04 |  |  |  |  |  |  |  |  |  |  |  |  |  |  |
| SM01 |  |  |  |  |  |  |  |  |  |  |  |  |  |  |
| SM02 | 1.000 |  |  |  |  |  |  |  |  |  |  |  |  |  |
| SM03 | 0.070 | 1.000 |  |  |  |  |  |  |  |  |  |  |  |  |
| SM04 | 0.119 | 0.981 | 1.000 |  |  |  |  |  |  |  |  |  |  |  |
| SM05 | 0.270 | 0.465 | 0.553 | 1.000 |  |  |  |  |  |  |  |  |  |  |
| SM06 | 0.036 | 0.968 | 0.948 | 0.337 | 1.000 |  |  |  |  |  |  |  |  |  |
| SM07 | -0.027 | -0.018 | -0.012 | -0.013 | -0.010 | 1.000 |  |  |  |  |  |  |  |  |
| SM08 | 0.082 | 0.959 | 0.950 | 0.385 | 0.965 | -0.013 | 1.000 |  |  |  |  |  |  |  |
| SM09 | -0.039 | -0.042 | -0.047 | -0.065 | -0.041 | 0.982 | -0.034 | 1.000 |  |  |  |  |  |  |
| SM10 | 0.037 | 0.945 | 0.916 | 0.282 | 0.964 | -0.020 | 0.987 | -0.031 | 1.000 |  |  |  |  |  |
| SM11 | -0.041 | -0.039 | -0.043 | -0.063 | -0.034 | 0.856 | -0.034 | 0.915 | -0.034 | 1.000 |  |  |  |  |
| SM12 | 0.091 | 0.941 | 0.937 | 0.397 | 0.939 | -0.017 | 0.989 | -0.035 | 0.974 | -0.033 | 1.000 |  |  |  |
| SM13 | -0.036 | -0.039 | -0.045 | -0.061 | -0.039 | 0.884 | -0.031 | 0.941 | -0.029 | 0.994 | -0.028 | 1.000 |  |  |
| SM14 | 0.934 | 0.158 | 0.227 | 0.379 | 0.125 | -0.008 | 0.183 | -0.039 | 0.116 | -0.031 | 0.210 | -0.029 | 1.000 |  |
| SM15 | 0.316 | 0.648 | 0.727 | 0.676 | 0.583 | -0.017 | 0.704 | -0.054 | 0.622 | -0.053 | 0.735 | -0.040 | 0.453 | 1.000 |
